# Cell-Specific Regulation of Inflammatory Cytokines and Acute-Phase Proteins by the Glucocorticoid Receptor

**DOI:** 10.1155/2023/4399998

**Published:** 2023-11-28

**Authors:** Rebecca Winkler, Hong Lu

**Affiliations:** Department of Pharmacology, SUNY Upstate Medical University, Syracuse, NY 13210, USA

## Abstract

**Background:**

Literature and data mining found abnormal induction of chemokine (C-X-C motif) ligand 1 (CXCL1) and CXCL8 and down-regulation of CXCL2 in inflammatory liver diseases. This study was performed to understand the glucocorticoid receptor's (GR's) effects on chemokine and acute-phase protein expression in human liver, in settings of bacterial infection (modeled using LPS) or inflammation (modeled using TNF*α*).

**Methods:**

Primary human hepatocytes (PHH) were treated with combinations of tumor necrosis factor alpha (TNF*α*), lipopolysaccharide (LPS), and dexamethasone (DEX) for 24 h, following which chemokine mRNA and protein expression were analyzed using qPCR and enzyme-linked immunosorbent assay assays. Dual luciferase assays were performed on transfected cell lines. Mutant CXCL2 promoters were used in dual luciferase assays to identify specific regions of the CXCL2 promoter affected by GR, TNF*α*, or hepatocyte nuclear factor 4*α* (HNF4*α*, a liver-enriched transcription factor).

**Results:**

In PHH from donor 1, GR strongly inhibited LPS-induced CXCL1 and CXCL8 translation and transcription, whereas CXCL2 transcription tended to increase with DEX treatment. In PHH from donor 2, DEX treatment inhibited protein expression and secretion of CXCL1 and CXCL8 induced by TNF*α* and/or LPS, whereas CXCL2 upregulation was largely unaffected by DEX treatment. In nonliver HEK293T cells GR activity inhibited CXCL2 promoter activity. However, in liver-derived HEPG2 cells, GR induced CXCL2 promoter activity. A 407-base pair region upstream of CXCL2 promoter is necessary for full GR functionality in HEPG2 cells. TNF*α* synergized with HNF4*α* in inducing CXCL2 promoter activity in HEPG2 cells.

**Conclusions:**

GR's effects on chemokine expression are cell-type specific and chemokine specific. GR down-regulated CXCL1 and CXCL8 in different cell types, whereas the specific activation of CXCL2 in hepatocytes and down-regulation of CXCL2 in nonhepatocytes by GR appears due to cell-specific utilization of CXCL2 promoter. By specifically increasing GR activity in the liver, we may normalize chemokine imbalances and prevent sepsis in inflammatory liver diseases.

## 1. Introduction

An inflammatory response is crucial to properly address a bacterial invasion or mechanical trauma. However, an exaggerated and unchecked systemic inflammatory response to infections results in self-inflicted damage defined as sepsis. An important step in preventing this life-threatening overcompensation into sepsis is to maintain appropriate levels and ratios of cytokines [1].

Chemokines, a subset of cytokines, are the biological equivalent of emergency flares, labeling trouble zones, and recruiting assistance from passing immune cells. They can be secreted by white blood cells requesting backup from other white blood cells, or they can be secreted by the injured or inflamed tissue itself. As such, chemokines are often, although not always, considered proinflammatory molecules [2]. Although the chemokine family is large, comprising more than 50 members [3], we limited our study to a few highly altered chemokines, focusing in particular on some of the ones affecting primarily neutrophils: C-X-C motif chemokine ligands 1, 2, and 8 (CXCL1, CXCL2, and CXCL8), working through the C-X-C motif chemokine receptors 1 and/or 2 (CXCR1 and/or CXCR2). Also briefly mentioned are C-X-C motif chemokine ligand 10 (CXCL10), which targets T-cells via the C-X-C motif chemokine receptor 3 (CXCR3), and C-C motif chemokine ligand 2 (CCL2), which recruits monocytes and macrophages using C-C motif chemokine receptor 2 [3]. Besides their involvement with white blood cells, chemokines can act as paracrine or autocrine ligands influencing fibrosis, apoptosis, and cell survival, proliferation, and angiogenesis [3].

The most common class of medication used to treat inflammation is glucocorticoids (GCs), including the natural endogenous form cortisol and several synthetic forms, such as prednisone, prednisolone, and dexamethasone (DEX). GCs are frequently utilized in the clinical setting for inflammatory conditions such as asthma, arthritis, autoimmune diseases, hives, alcoholic hepatitis (AH), sepsis, and so forth. We set out to investigate GC effects on chemokine and acute-phase protein (APP) expression in the liver, an organ with a front-and-center role in the delicate art of balancing infection and inflammation [4].

The liver produces a number of chemokines [3], neutralizes or removes toxins, and hosts neutrophils that snare blood-born bacteria [5]. The liver also produces an assortment of proteins known as APPs which are secreted into the serum to flag bacteria for destruction by white blood cells, sequester available iron, regulate blood coagulation, and perform a variety of other infection- and inflammation-management functions [6]. Dysregulated chemokines in the liver are associated with liver problems such as AH [7], nonalcoholic steatohepatitis (*Supplementary 1*), or with transplant-induced ischemia reperfusion injury (IRI) [8] (*Supplementary 1*). Since the liver is so crucial to a proper immune and inflammatory response, patients with liver diseases are especially vulnerable to developing sepsis [9, 10].

We hypothesize that specifically activating the glucocorticoid receptor (GR) in the liver will help to rebalance dysregulated hepatic chemokines and APPs in patients at high risk of sepsis. In this study, we employed qPCR and enzyme-linked immunosorbent assay (ELISA) of primary human hepatocytes (PHH) to analyze the effects of DEX, tumor necrosis factor alpha (TNF*α*), and/or lipopolysaccharide (LPS) on CXCL1, CXCL2, and CXCL8 mRNA and protein expressions, also comparing secretion profiles of these chemokines. Several other genes and/or proteins were analyzed as well, including CXCL10, plasminogen activator inhibitor 1 (PAI-1, also known as SERPINE1), hepcidin (HAMP), serum amyloid A1 (SAA1), CD163 molecule (CD163), interleukin 1 beta (IL1B), and CCL2. We used dual luciferase assays to compare the promoter activities of CXCL1, CXCL2, CXCL8, and CXCL10 in two different cell lines following treatment with DEX and/or TNF*α*, and we used site-directed mutagenesis of the CXCL2 promoter to determine in closer detail the unusual regulation seen with this chemokine in particular.

## 2. Materials and Methods

### 2.1. Primary Cell Culture

PHH from live donors were isolated and shipped by the Liver Tissue and Cell Distribution System of the National Institute of Diabetes and Digestive and Kidney Diseases (University of Pittsburgh, PA). Patient 1 was a 35-year-old female with a hemangioma, whereas patient 2 was a 63-year-old male with colorectal cancer metastasized to the liver. Upon arrival, culture media was replaced with serum-free media (DMEM/F12 supplemented with trace elements, bovine serum albumin, insulin, glucagon, transferrin, and penicillin/streptomycin, modified from [11, 12]) and cultured overnight. The following day the media was replaced with media including TNF*α*, LPS, DEX, and/or the GR antagonist RU486, with dimethyl sulfoxide (DMSO) concentrations of 0.1% in all groups of the experiments. After culturing cells with drugged media for 24–26 hr, an aliquot of media was collected for ELISA assay, and then the remaining media was removed and cells were frozen at −80°C.

### 2.2. Study of Effects of GCs on LPS-Stimulated Induction and Release of Cytokines from Human Whole Blood

Aliquots of 0.24 mL fresh heparinized human whole blood (WB) from a healthy volunteer (IRB#754811-13) were added to a 1 : 1 ratio of RPMI-1640 medium that contained drugs and 2 ng/mL LPS and incubated for 4 hr (at 37°C × 250 rpm). After 4 hr incubation with LPS and/or DEX, samples were centrifuged at 1,500 g for 10 min at 25°C and supernatant stored at −80°C for analysis of cytokines by ELISA. Total RNAs were prepared from the lower layer of blood cells using the RiboPure RNA Purification kit, blood (AM 1928, Invitrogen) for qPCR determination of mRNA expression of cytokines.

### 2.3. qPCR Quantification of mRNA Expression

RNA extraction from PHH was carried out using RNA STAT-60 (Tel-Test, Inc.) or a combination of RNA STAT-60 in combination with Direct-zol RNA MiniPrep Plus (Zymo Research, Cat. No.: R2072). RNA was quantified via NanoDrop and reverse transcribed using iScript cDNA Synthesis Kit from Bio-Rad. qPCR was performed using CFX Maestro Version 4.1.2433.1219 with a 4-cycle touchdown at the beginning of the protocol to improve detection sensitivity and amplification efficiency [13]. Primers were designed, when possible, to include a large intron region to avoid amplifying any contaminating DNA, and a melt curve was employed at the end of the protocol to identify any off-target or primer-dimer amplification. In most cases, samples were run in duplicates and the average of the technical replicates was used for graphing and statistics. Gene expression was normalized using AKIRIN1 as a housekeeping gene [14], using the formula power (2,(AKIRIN1 cQ—target cQ)), and plotted relative to DMSO controls. See supplemental for a list of primer pairs.

### 2.4. ELISA Assays

ELISA assays were performed using kits from R&D systems (bio-techne).

1. Human CXCL1/GRO alpha DuoSet ELISA (catalog number DY275-05)
2. Human CXCL2/GRO beta DuoSet ELISA (catalog number DY276-05)
3. Human IL-8/CXCL8 DuoSet ELISA (catalog number DY208-05)
4. Human CXCL10/IP-10 DuoSet ELISA (catalog number DY266-05)
5. Human Total Serpin E1/PAI-1 DuoSet ELISA (catalog number DY9387-05)
6. Human Hepcidin DuoSet ELISA (catalog number DY8307-05).

ELISA assays were performed on culture media for both hepatocytes from donor 1 and donor 2. ELISA assay of cellular lysates was performed from donor 2. Before lysing PHH in radioimmunoprecipitation assay buffer (RIPA) buffer (Alfa Aesar by Thermo Fisher Scientific, J62885 RIPA buffer with Triton® X-100), scrapings were taken from each well while frozen for RNA extraction. Afterward, remaining cells were lysed with RIPA buffer and protease/phosphatase inhibitor (Halt™ Protease and Phosphatase Inhibitor Cocktail, Catalog number: 78442, Thermo Fisher Scientific). Lysates were vortexed, incubated on ice for approximately 1–2 hr, then frozen at −80°C. Before ELISA assays, PHH lysates were vortexed, sonicated 3x, and centrifuged 14,000 g for 10 min at 4°. Protein concentration of PHH lysates was determined using DC Protein Assay (Bio-Rad, cat# 500-0116), and cell lysates were diluted to 1 mg/mL. ELISA results were quantified using a BioTek Synergy H1 microplate reader (run with Gen5 2.09 software from BioTek Instruments, Inc). Standard and sample absorbances were read at 450 nm, and background absorbances at 540 or 570 nm were subtracted from each well per protocol. Standard curves were plotted in Microsoft Excel, and absorbance readings were converted to chemokine concentrations based on the standard curve and each sample's dilution factor. CXCL2 media from patient 2 was plotted against a standard curve calculated using absorbances at both 450 and 455 nm due to the highest concentration standard being above the detection limit at 450 nm alone.

### 2.5. Plasmid Construction, Transient Transfection, and Dual Luciferase Assays

See supplemental for a list of plasmids. The reporter vectors for promoters of human CXCL1, CXCL2, CXCL8, and CXCL10 were generated by PCR cloning into the KpnI/MluI sites of pGL3-basic vector (Promega) using total DNA from HEK293 cells as the template. Transcription factor binding sites were identified/predicted using PROMO version 3.0 at the ALGGEN server [15, 16]. Reporter vectors for mutated/deleted human CXCL2 promoter were generated using the Q5® Site-Directed Mutagenesis Kit (New England BioLabs). A secondary reporter vector, pRL-Basic was generated by replacing the firefly luciferase cDNA in the pGL3-Basic vector with the Renilla luciferase cDNA. Plasmids were grown in *Escherichia coli* (Q5 Site-Directed Mutagenesis Kit E0554S from New England BioLabs) and purified using either Zyppy Plasmid Miniprep Kit (D4020 from Zymo Research), GeneJET Plasmid Miniprep Kit (K0503 from Thermo Scientific) or PureLink HiPure Plasmid Midiprep Kit (K210004 from Invitrogen by Life Technologies).

Human hepatoma HEPG2 and human embryonic kidney 293T (HEK293T) cells (ATCC) were grown in DMEM with 9% fetal bovine serum (FBS) and treated with penicillin/streptomycin. HEPG2 cells were plated for luciferase assay at a concentration of 3 × 10^4^ cells per well of a 96-well plate, and HEK293T cells at 1.25 × 10^4^ per well. Transfections of HEPG2 or HEK293T cells were performed using Lipofectamine 3000 (Invitrogen by Thermo Fisher Scientific L3000-015). Plasmids included pGL3 vectors for quantifying promoter activity, pRL-basic vector for normalization, a GR expression vector pK7GR (with the EGFP tag removed from the pk7-GR-GFP vector, #15534, Addgene) and HNF4A expression vectors [17], and a PCMX vector and a fluorescent vector to equalize plasmid concentrations at 95–100 ng per well and monitor transfection efficiency. Following transfection, the cells were incubated overnight, then treated with DEX (or DMSO control) and/or TNF*α* for 24 hr. Media was then removed and plates were stored at −80°C until being assayed. Dual Luciferase assays were performed (kit E1980 from Promega) using a 6-s protocol. Background was subtracted, and the promoter reporter activities were normalized to the Renilla luciferase activity of pRL-basic, with the control values set as 1.0.

### 2.6. Statistics

For qPCR data, in most cases samples were run in duplicate with the average of the technical replicates used for graphing and statistics. Gene expression was normalized using AKIRIN1 as a housekeeping gene [14], log2 transformed in Excel, and then plotted and analyzed relative to DMSO controls. The qPCR, ELISA, and dual luciferase assays were assessed in Graph Pad Prism (version 9.5.1 for Windows; Graph Pad Software, San Diego, CA, USA, https://www.graphpad.com) with Brown–Forsythe and Welch ANOVA tests and Dunnett T3 post hoc testing of selected pairings as shown. Although in some instances Shapiro–Wilk testing identified possible violations of the normality assumption for particular groups, given the small *n* of 3–4 replicates or wells per group, nonparametric testing did not seem appropriate. Data that were log10 transformed before statistical analysis are notated in the figure legends. The data mining results from GSE17470 (shown in supplemental) were assessed in Graph Pad Prism using the Mann–Whitney test, Welch's *t*-test, and unpaired *t*-test as appropriate per Shapiro–Wilk normality testing and similarity of standard deviations between control and test groups. Data mining from GSE151648 was analyzed using the Wilcoxon matched-pairs signed rank test to compare matched pre- and post-transplant samples and the Mann–Whitney test to compare patients without IRI post-transplant to patients with IRI.

## 3. Results

### 3.1. GR Altered CXCL1, CXCL2, and CXCL8 Transcription and Upregulation by TNF*α* or LPS in PHH

To determine the regulation of chemokine expression in PHH, we compared the effects of DEX on PHH in the setting of generic inflammation versus bacterial infection, mimicked by the treatment with TNF*α* and LPS. TNF*α* is an endogenous protein rapidly produced by immune cells [18] in response to a variety of infectious and noninfectious assaults such as IRI [19], and is known to induce CXC chemokines in the liver [20]. Under normal conditions, LPS is readily removed by Kupffer cells and sinusoidal endothelial cells in the liver [21], and thus hepatocytes have very little exposure to LPS during the early stage of infections. Thus, a large increase in PHH exposure to LPS occurs in the late/severe stage of bacterial infections when hepatic LPS clearance by Kupffer cells and sinusoidal endothelial cells has been compromised/saturated. PHH were cotreated with DMSO, DEX 0.1 or 1 *µ*M, TNF*α* 50 ng/mL, and/or LPS 10 ng/mL for 24 hr.

**Figure 1:**
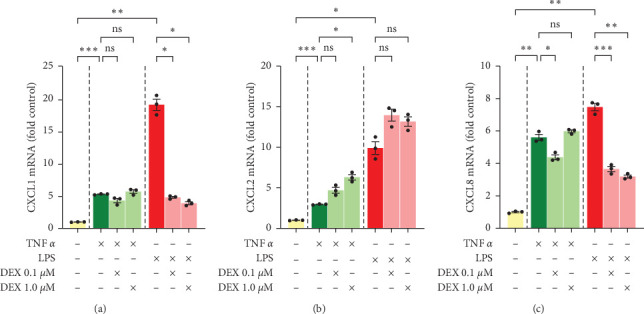
Chemokine mRNA levels in primary human hepatocytes, donor 1. Cells were treated with TNF*α* 50 ng/mL, LPS 10 ng/mL, and/or dexamethasone 0.1 or 1 *µ*M for 24 hr. Gene expression for (a) CXCL1, (b) CXCL2, and (c) CXCL8 was assessed by qPCR and normalized to AKIRIN1. Mean ± SE, *n* = 3 replicate wells. *⁣*^*∗*^*p* ≤ 0.05, *⁣*^*∗*^*⁣*^*∗*^*p* ≤ 0.01, and *⁣*^*∗*^*⁣*^*∗*^*⁣*^*∗*^*p* ≤ 0.001. ns, not significantly different.

The qPCR results showed that CXCL1 mRNA expression was induced 5-fold by TNF*α* treatment but relatively unaffected by the addition of DEX (Figure 1(a)). CXCL2 mRNA expression was induced 3-fold by TNF*α* and further increased up to 6-fold by the addition of DEX (Figure 1(b)). CXCL8 expression was induced 5-1/2 fold by TNF*α*, which was modestly inhibited by cotreatment with a low-dose DEX (0.1 *µ*M) (4-fold baseline) but not affected (6-fold baseline) by the higher dose of 1 *µ*M DEX (Figure 1(c)).

Although the effects of TNF*α* and LPS on chemokine expressions will, of course, differ due to differences in dosages, pathways, and biological sources; assessing GC response in these two distinct and sometimes overlapping settings is important to understanding chemokine regulation in the liver. LPS, like TNF*α*, dramatically induced mRNA expression of CXCL1, CXCL2, and CXCL8 in PHH (Figure 1). CXCL1 increased the most following 10 ng/mL LPS treatment (Figure 1(a)), with mRNA levels rising 19-fold baseline control level. LPS treatment increased CXCL2 (Figure 1(b)) and CXCL8 (Figure 1(c)) mRNAs 10- and 7-fold, respectively. CXCL2 and CXCL8 levels varied drastically with cotreatment of LPS and DEX, however. Both CXCL1 and CXCL8 induction were markedly inhibited by DEX, with mRNA levels rising to only 3- to 5-fold baseline when LPS treatment was combined with DEX. CXCL2, on the other hand, showed no decrease at all with DEX cotreatment and even continued its upward trend to 14-fold control (Figure 1(b)).

To summarize, both TNF*α* and LPS increased mRNA expression of CXCL1, CXCL2, and CXCL8. DEX strongly counteracted LPS-induced CXCL1 and CXCL8 transcription but had little to no effect on TNF-induced transcription of these two chemokines in PHH. DEX tended to amplify rather than inhibit TNF- and LPS-induced transcription of CXCL2.

### 3.2. DEX Affected Chemokine Secretion Differently than Chemokine Transcription in PHH

**Figure 2:**
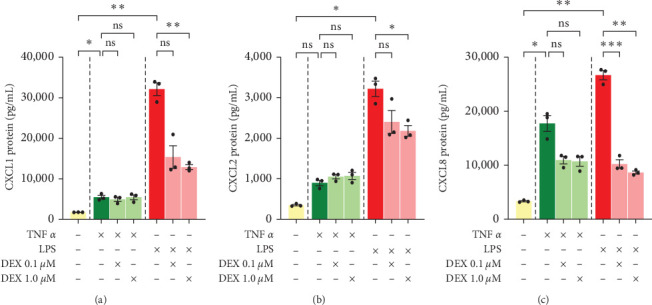
Chemokine protein levels in culture media of primary human hepatocytes, donor 1. ELISA assays of (a) CXCL1, (b) CXCL2, and (c) CXCL8 were done on cell culture media collected after 24-h incubation of cells treated with TNF*α* 50 ng/mL, LPS 10 ng/mL, and/or dexamethasone 0.1 or 1 *µ*M for 24 hr. Mean ± SE, *n* = 3 replicate wells. *⁣*^*∗*^*p* ≤ 0.05, *⁣*^*∗*^*⁣*^*∗*^*p* ≤ 0.01, and *⁣*^*∗*^*⁣*^*∗*^*⁣*^*∗*^*p* ≤ 0.001. ns, not significantly different.

Since changes in transcription do not necessarily correlate directly with changes in translation, protein stability, or secretion, we performed ELISA assay on the PHH culture media to assess DEX effect on secreted chemokine levels. Secreted levels of CXCL1, CXCL2, and CXCL8 for the most part resembled mRNA levels in PHH, with TNF*α* and LPS both inducing chemokine secretion and DEX inhibiting LPS- but not TNF-induced effects (Figure 2).

A few differences were observed, however. First, secreted protein levels of CXCL1 were higher in LPS-DEX combinations (7- to 9-fold basal level) than TNF-DEX combinations (3-fold basal level) (Figure 2(a)), despite nearly identical mRNA expressions between these two groups (4–5- vs. 4–6-fold basal mRNA expression) (Figure 1(a)). Second, secreted protein levels of CXCL2 showed a slightly different response to DEX treatment than mRNA levels would have suggested. Although mRNA levels of CXCL2 were higher with DEX + TNF*α* than TNF*α* alone (Figure 1(b)), secreted protein levels were very similar between the two groups (at 2-1/2-fold vs. 3-fold) (Figure 2(b)). In addition, a trend of increased CXCL2 mRNA was seen with DEX being added to LPS (Figure 1(b)), a statistically significant decrease in secreted CXCL2 protein was identified in DEX 1 *µ*M + LPS compared to LPS alone (LPS being 9-fold control, dropping to 6- or 7-fold control by DEX) (Figure 2(b)). This raises the question of whether CXCL2 protein may, in fact, be increased but not secreted into the media in these groups. Third, TNF-induced secretion of CXCL8 tended to be blunted by both doses of DEX (5-fold blunted to 3-fold control) (Figure 2(c)), whereas only low-dose DEX resulted in a decrease of CXCL8 mRNAs (Figure 1(c)). Thus, DEX affected CXCL8 secretion through more than just transcriptional changes.

In summary, both TNF*α* and LPS treatment resulted in increased chemokine secretion to the media, as expected from the qPCR results. DEX inhibited LPS-induced CXCL1 and CXCL8 transcription and secretion but had little to no effect on inhibiting TNF-induced CXCL1 and CXCL8 transcription and secretion in PHH. The DEX-related trend of amplification of CXCL2 mRNA induced by TNF*α* and LPS was largely lost or even reversed when considering protein secretion. Thus, mRNA changes induced by DEX largely but imperfectly predict changes in chemokine secretion.

### 3.3. Transcription and Secretion of CXCL10, PAI-1, and HAMP were Affected by DEX

**Figure 3:**
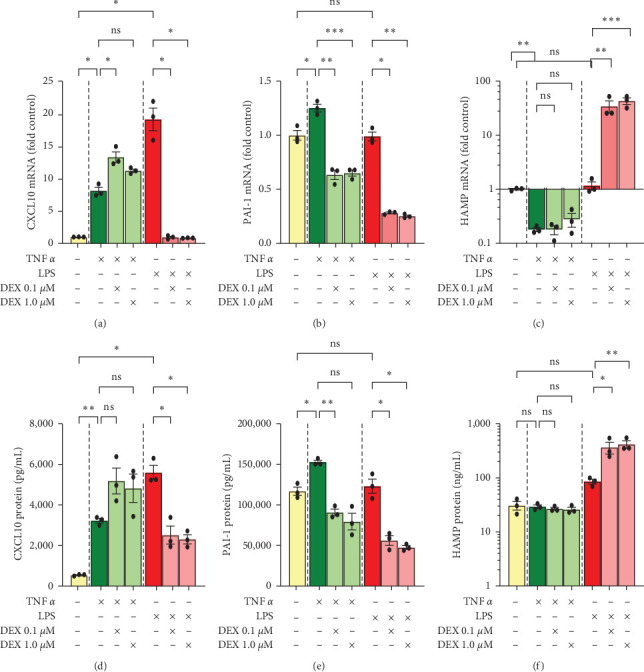
Cellular mRNA and corresponding culture-media protein levels, donor 1. Cells were treated with TNF*α* 50 ng/mL, LPS 10 ng/mL, and/or dexamethasone 0.1 or 1 *µ*M for 24 hr. Gene expressions of (a) CXCL10, (b) PAI-1, and (c) HAMP were assessed by qPCR and normalized to AKIRIN1 mRNA. Corresponding protein levels (d–f) were assessed by ELISA assays of culture media. Data for HAMP (Hepcidin) was log10 transformed for statistical analysis and graphing. Mean ± SE, *n* = 3 replicate wells. *⁣*^*∗*^*p* ≤ 0.05, *⁣*^*∗*^*⁣*^*∗*^*p* ≤ 0.01, and *⁣*^*∗*^*⁣*^*∗*^*⁣*^*∗*^*p* ≤ 0.001. ns, not significantly different.

Although the main focus of this study was GR's effect on CXCL1, CXCL2, and CXCL8, we also analyzed the mRNA and protein expression of CXCL10, PAI-1, and HAMP (Figure 3).

CXCL10 transcription was upregulated by TNF*α* and LPS (8- and 19-fold, respectively). Interestingly, DEX treatment augmented TNF-induced transcription (from 8-fold up to 11- or 13-fold baseline) but dramatically downregulated LPS-induced transcription (from 19-fold baseline down to baseline levels) (Figure 3(a)). ELISA assay closely replicated qPCR data for TNF, with TNF*α* increasing 6-fold and addition of DEX increasing CXCL10 protein secretion to 9- or 10-fold. However, LPS increased CXCL10 protein secretion by 10-fold, and DEX decreased CXCL10 protein secretion to 4- or 5-fold baseline (Figure 3(d)) as opposed to the mRNA levels that returned to baseline.

Like the chemokines CXCL1, CXCL2, and CXCL8, PAI-1 (a.k.a. SERPINE1) also plays an important role in neutrophil migration [22]. In addition to its well-known inhibition of fibrinolysis, PAI-1 is also involved in cholesterol regulation [23]. PAI-1 showed little to no induction by either TNF*α* or LPS (Figure 3(b)). DEX, however, decreased both mRNA expression (to 60%–70% baseline with TNF*α* and 30% with LPS) (Figure 3(b)) as well as protein secretion of PAI-1 (to 70%–80% baseline with TNF*α* and 40%–50% with LPS) (Figure 3(e)). Interestingly, this drop in PAI-1 was particularly dramatic when DEX was combined with LPS despite the complete lack of response to LPS alone.

HAMP is crucial for properly regulating (decreasing) iron levels in the blood [24]. Increased iron aggravates ferroptosis in liver diseases and is strongly correlated with increased risk of severe bacterial infections [25, 26]. HAMP was dramatically downregulated by TNF*α* in terms of mRNA (to 20% of baseline) (Figure 3(c)), but this drop was not reflected in terms of protein secretion. ELISA assay identified similar concentrations of HAMP secreted with and without TNF*α* (Figure 3(f)). Moreover, DEX had no effect on HAMP transcription or secretion when combined with TNF. When combined with LPS, however, a sudden and dramatic upregulation was observed in both mRNA (up to 34- or 43-fold baseline with DEX + LPS) (Figure 3(c)) and protein levels (from 3-fold with LPS to 12- or 14-fold with addition of DEX) (Figure 3(f)).

### 3.4. Transcriptional Changes were Observed in Other Genes as well

**Figure 4:**
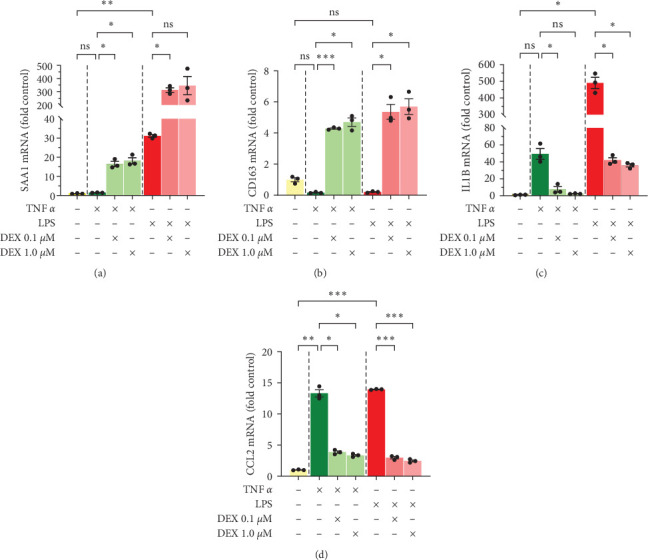
Cellular mRNA levels in primary human hepatocytes, donor 1. Cells were treated with TNF*α* 50 ng/mL, LPS 10 ng/mL, and/or dexamethasone 0.1 or 1 *µ*M for 24 hr. Gene expressions of (a) SAA1, (b) CD163, (c) IL1B, and (d) CCL2 were assessed by qPCR and normalized to AKIRIN1. Mean ± SE, *n* = 3 replicate wells. *⁣*^*∗*^*p* ≤ 0.05, *⁣*^*∗*^*⁣*^*∗*^*p* ≤ 0.01, and *⁣*^*∗*^*⁣*^*∗*^*⁣*^*∗*^*p* ≤ 0.001. ns, not significantly different.

Some other genes also analyzed by qPCR included two proteins known to be involved in the acute-phase response, SAA1 and CD163, and the cytokines IL1B and CCL2 (Figure 4). SAA1 increased dramatically with the combination of TNF*α* and DEX (up to 18-fold) or LPS and DEX (up to 346-fold) (Figure 4(a)). For CD163, treatment with TNF*α* or LPS tended to decrease (*p*=0.07and0.08, respectively) mRNA expression to 20% baseline, whereas addition of DEX counteracted this loss, even increasing expression above baseline (5- and 6-fold, respectively) (Figure 4(b)). For both cytokines IL1B and CCL2, TNF*α* or LPS increased expression: TNF*α* increased IL1B to 49-fold (*p*=0.05), and LPS increased it to 490-fold (Figure 4(c)); TNF*α* increased CCL2 to 13-fold, LPS increased it to 14-fold (Figure 4(d)). DEX strongly counteracted the induction of IL1B and CCL2 by TNF*α* and LPS, with IL1B dropping to a low of 2-fold baseline with TNF*α* and a low of 36-fold with LPS (Figure 4(c)), and CCL2 dropping to approximately 3-fold with either TNF*α* or LPS (Figure 4(d)).

### 3.5. Chemokine Expression in Response to TNF*α*, LPS, and DEX were Patient Specific but Generally Repeatable

To further validate our findings and account for patient-specific responses, we obtained a second batch of PHH from the University of Pittsburgh. This time PHH were treated with DMSO control or 1 *µ*M DEX (the high dose from the earlier PHH study), TNF*α* 50 ng/mL, LPS 10 ng/mL (both the same as Donor 1), or RU486 (a GR antagonist) 5 *µ*M combined with DEX 1 *µ*M as a negative control for GR-dependent effects.

**Figure 5:**
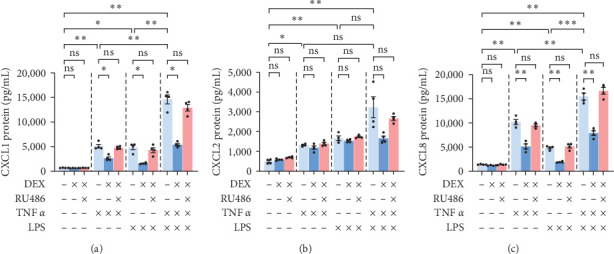
Chemokine protein levels in culture media of primary human hepatocytes, donor 2. Cells were treated with TNF*α* 50 ng/mL, LPS 10 ng/mL, dexamethasone 1 *µ*M, and RU486 5 *µ*M for 24 hr before media collection for ELISA assays of (a) CXCL1, (b) CXCL2, and (c) CXCL8. CXCL2 was log10 transformed for statistical analysis due to the large error bar in the TNF-LPS group. Mean ± SE, *n* = 4-well replicates, and *⁣*^*∗*^*p* ≤ 0.05, *⁣*^*∗*^*⁣*^*∗*^*p* ≤ 0.01, and *⁣*^*∗*^*⁣*^*∗*^*⁣*^*∗*^*p* ≤ 0.001. ns, not significantly different.

In comparison to the first patient, PHH from this second patient was much more resistant to LPS treatment, manifested as much less increase of LPS-stimulated secretion of chemokines CXCL1, CXCL2, and CXCL8 (Figure 5). It is worth noting that while the first batch of PHH was obtained from a patient with a hemangioma, this next batch of PHH was obtained from a patient with metastatic colorectal cancer. It has been reported in a recent publication by de Waal et al. [27] that there is a direct correlation between colorectal cancer and elevated LPS levels. Thus, it is possible that these PHH were already exposed to high LPS levels in situ and developed a degree of LPS tolerance when compared with the hepatocytes procured from a nonmalignant environment.

Despite the blunted LPS response, these hepatocytes, like the first batch of PHH, demonstrated increased levels of secreted chemokines for CXCL1, CXCL2, and CXCL8 when treated with TNF*α* or LPS. CXCL1 increased 7-fold with TNF*α* and 7-fold with LPS (Figure 5(a)). CXCL2 increased 3-fold with TNF*α* and 4-fold with LPS (Figure 5(b)). CXCL8 increased 7-fold with TNF*α* and 3-fold with LPS (Figure 5(c)). DEX treatment had no effect on basal chemokine secretion but attenuated LPS and TNF*α* response for CXCL1 and CXCL8: CXCL1 with TNF*α* went from 7- to 4-fold upon addition of DEX, with LPS went from 7- to 2-fold with DEX (Figure 5(a)); CXCL8 with TNF*α* went from 7-fold without DEX to 4-fold with DEX, and LPS went from 3-fold to just above baseline with DEX (Figure 5(c)). Like the first PHH, DEX had no effect on TNF-induced CXCL2 secretion (Figure 5(b)). This time DEX had no effect on LPS-induced secretion either.

The combination of LPS and TNF*α* dramatically increased the secretion of all three chemokines to levels higher than either treatment alone, and DEX counteracted this extreme increase in all three cases. CXCL1 with TNF*α* = 7-fold baseline, with LPS = 7-fold, with combination = 21-fold, with combination + DEX = 8-fold (Figure 5(a)). CXCL2 with TNF*α* = 3-fold, with LPS = 4-fold, with combination = 6-fold, with combination + DEX = 4-fold (Figure 5(b)). CXCL8 with TNF*α* = 7-fold, with LPS = 3-fold, with combination = 11-fold, with combination + DEX = 6-fold (Figure 5(c)). Treatment with RU486 abolished the inhibitory effects of DEX on LPS- and/or TNF-increased chemokine secretion, further validating the role of GR in these antagonizing effects.

### 3.6. CXCL1, CXCL2, and CXCL8 Showed Different Cell-Association and Secretion Profiles

Since there were some differences between the qPCR data and the ELISA data (e.g., CXCL2 mRNA increasing with DEX treatment but the secretion of CXCL2 protein either stable or dropping for the same groups), we next asked whether chemokine protein levels were changing, perhaps through changes in translation or protein stability, or just chemokine localization (cell-associated vs. secreted) was changing. To answer this question, we used RIPA buffer to lyse the PHH and then repeated the ELISA assays using cellular lysates to compare with the results from the culture media.

**Figure 6:**
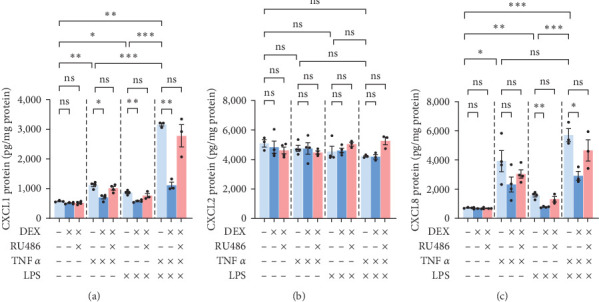
Chemokine protein levels in cellular lysates of primary human hepatocytes, donor 2. Cells were treated with TNF*α* 50 ng/mL, LPS 10 ng/mL, dexamethasone 1 *µ*M, and RU486 5 *µ*M for 24 hr, frozen, then lysed in RIPA buffer for ELISA assays of (a) CXCL1, (b) CXCL2, and (c) CXCL8. CXCL8 data was log10 transformed for statistical analysis due to large error bars. Mean ± SE, *n* = 3–4-well replicates, and *⁣*^*∗*^*p* ≤ 0.05, *⁣*^*∗*^*⁣*^*∗*^*p* ≤ 0.01, and *⁣*^*∗*^*⁣*^*∗*^*⁣*^*∗*^*p* ≤ 0.001. ns, not significantly different.

ELISA assay of CXCL1 cellular lysates replicated the trends of the ELISA assay of the culture media; however, the range of values was much tighter in the lysates compared to the media, with a maximum value of 6-fold baseline in the cell lysates (Figure 6(a)) but 21-fold in the media (Figure 5(a)). CXCL2 was quite stable in the cellular lysates, with very little difference between any of the groups (Figure 6(b)) despite obvious differences in the culture media (Figure 5(b)). CXCL8 showed nearly identical trends of changes comparing cell lysates (Figure 6(c)) and culture media (Figure 5(c)) data.

**Figure 7:**
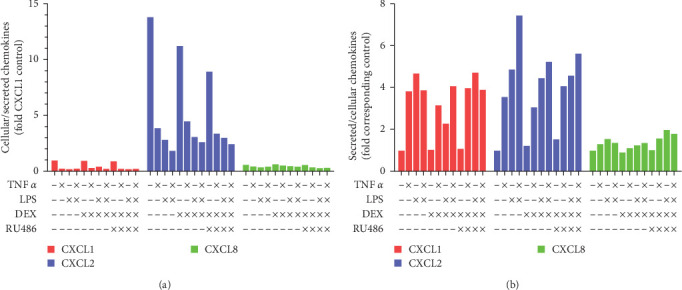
Comparisons of chemokine localization in cultured primary human hepatocytes, donor 2. Cells were treated with TNF*α* 50 ng/mL, LPS 10 ng/mL, dexamethasone 1 *µ*M, and RU486 5 *µ*M for 24 hr; and then the cell lysates and media were used for ELISA assays of chemokines. Data from Figures 5to 6 were used for comparisons of (a) cellular-association-to-secretion ratios relative to CXCL1 control. (b) Secretion-to-cellular-association ratios relative to respective chemokine control.

We next decided to display the data as a ratio of cell-associated to secreted. Since only a portion of cells in each well was sampled to prepare the cell lysates and the units differed (weight/mg protein for cell-associated vs. weight/mL for secreted), we divided each ratio by the CXCL1 control ratio, thus setting all values relative to baseline CXCL1 and eliminating the effects of differences in cell sampling. Compared to CXCL1 control, CXCL2 control was 14 times more cell-associated (Figure 7(a)). CXCL8 control, on the other hand, was less cell-associated than CXCL1 (60% CXCL1) (Figure 7(a)). When each chemokine was set relative to its own control rather than CXCL1′s, it became obvious that CXCL8 was the least sensitive of the three chemokines to localization changes due to TNF*α* or LPS: TNF and LPS markedly increased the ratio of secreted versus cell-associated CXCL1 and CXCL2 but barely affected the ratio of secreted versus cell-associated CXCL8 (Figure 7(b)).

### 3.7. DEX's Influence on CXCL2 was Cell-Type Specific

**Figure 8:**
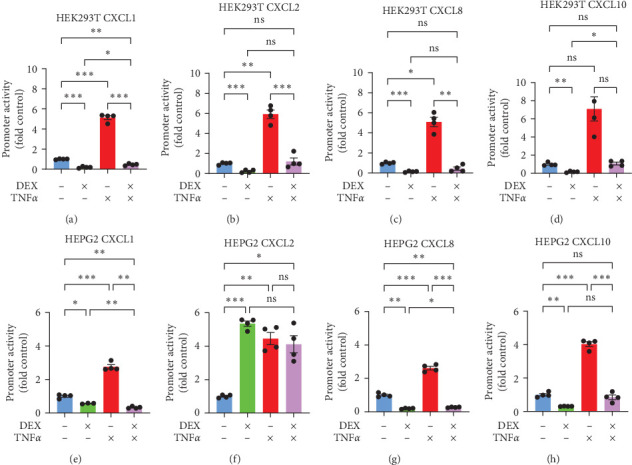
Dual luciferase assays of chemokine promoter activities in HEPG2 and HEK293T cell lines. HEPG2 (a–d) and HEK293T (e and f) cells were transfected with luciferase reporter plasmids for promoters of CXCL1 (a and e), CXCL2 (b and f), CXCL8 (c and g), and CXCL10 (d and h) and GR expression plasmid in dexamethasone-treated groups. Cells were incubated with plasmids overnight and then treated with dexamethasone 10 nM, TNF*α* 20 ng/mL, and/or DMSO (0.1%) control. Promoter activities were quantified by dual-luciferase assay 24 hr after drug treatment. Mean ± SE, *n* = 3–4-well replicates, and *⁣*^*∗*^*p* ≤ 0.05, *⁣*^*∗*^*⁣*^*∗*^*p* ≤ 0.01, and *⁣*^*∗*^*⁣*^*∗*^*⁣*^*∗*^*p* ≤ 0.001. ns, not significantly different.

It is well known that different cell types and organ systems may respond to pharmacologic or other stimulation in different ways. We found that in contrast to the trend of DEX potentiating TNF- and LPS-induced CXCL2 mRNA in PHH, DEX markedly down-regulated the LPS-induction of CXCL2, CXCL8, and IL1B mRNA in the human WB (*Supplementary 1*). We compared DEX's and TNF*α*'s effects on chemokine expression in two commonly used tumor cell lines (HEK293T and HEPG2) utilizing dual luciferase reporter assays. In the nonliver HEK293T cells, transfection of GR along with DEX treatment decreased promoter activities for CXCL1 to 19% baseline (Figure 8(a)), CXCL2 to 23% (Figure 8(b)), CXCL8 to 14% (Figure 8(c)), and CXCL10 to 16% (Figure 8(d)). The opposite was true with treatment of TNF*α*; promoter activity increased in all cases: 5-fold, 6-fold, 5-fold, and 7-fold (*p*=0.06 due to large error bar), respectively. GR activated by DEX counteracted TNF*α*, limiting TNF*α*-induced activation of all four chemokine promoters (CXCL1 to 46% baseline, CXCL2 returned to baseline, CXCL8 45% baseline, and CXCL10 returned to baseline). However, in HEPG2 cells, a hepatocellular carcinoma cell line, CXCL2 promoter was activated 5-fold rather than inhibited by GR and DEX (Figure 8(f)). This agrees with data from the PHH (Figure 1(b)). By contrast, the other three chemokine promoters (CXCL1, CXCL8, and CXCL10), all showed decreased activities in HEPG2 cells in response to GR and DEX (57% of baseline, 19%, and 31%, respectively) (Figures 8(e), 8(g), and 8(h)), as seen in the HEK293T cells. In addition, DEX-GR strongly activated the reporter for intron5–6 region of FKBP5 (*Supplementary 1*), a well-established GR target gene [28], confirming the transactivating capability of the transfected GR proteins in HEK293 cells. Thus, from the data we conclude that GR induces CXCL2 transcription in hepatocyte-type cells and decreases CXCL2 transcription in nonliver HEK293T cells.

### 3.8. Basal CXCL2 Promoter Activity Relied on an NF-*κ*B-Binding Region and a CRE-Binding Region

**Figure 9:**
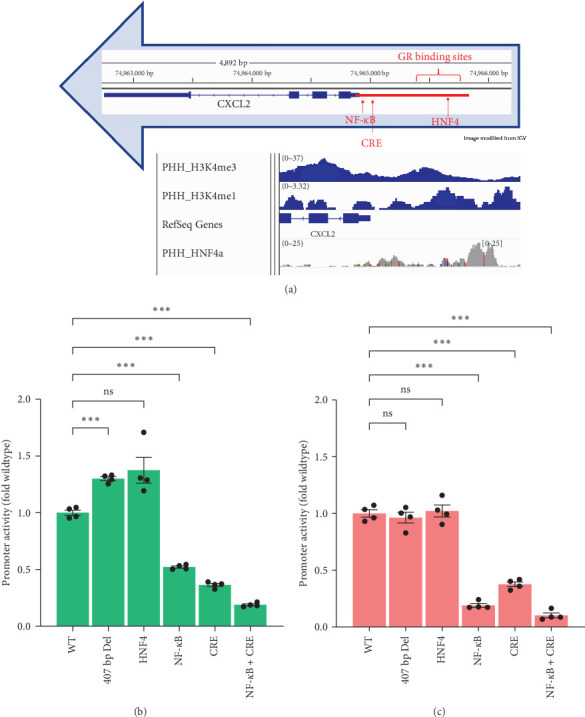
Basal reporter activities of wildtype and mutant CXCL2 promoters in HEPG2 and HEK293t cell lines. (a) Diagram of promoter (red bar) regions that were mutated or deleted, and data mining of ChIP-seq (GSE22078) of binding of HNF4*α* as well as histone marks trimethylation of lysine-4 of histone H3 (H3K4me3, GSM2533942) and monomethylation lysine-4 of histone H3 (H3K4me1, GSM2700191). The ChIP-seq data were retrieved from GEO DataSets and visualized in Integrative Genomics Viewer (IGV) [29, 30]. Promoter activities in (b) HEPG2 cells and (c) HEK293T cells were quantified by dual-luciferase assay after the transfected cells were treated with DMSO (0.1%) for 24 hr. (HEPG2 data is from the same experiment as Figure 10, graphed relative to WT plasmid control as opposed to DMSO.) Mean ± SE, *n* = 4 replicate wells per promoter. *⁣*^*∗*^*p* ≤ 0.05, *⁣*^*∗*^*⁣*^*∗*^*p* ≤ 0.01, and *⁣*^*∗*^*⁣*^*∗*^*⁣*^*∗*^*p* ≤ 0.001. ns, not significantly different.

To better understand the regulation of CXCL2 expression, we mutated/deleted several regions in the CXCL2 promoter (Figure 9(a)). Multiple binding sites for cAMP response elements (CRE), nuclear factor kappa B (NF-*κ*B), calcineurin/nuclear factor of activated T cells (NFAT), GR, and hepatocyte nuclear factor 4*α* (HNF4*α*) were predicted by PROMO [15, 16] in the CXCL2 promoter. HNF4*α* is a liver-enriched transcription factor that plays a critical role in regulating liver development and liver-specific gene expression [31]. Nucleotide substitutions were made to mutate the predicted binding sites for CRE, NF-*κ*B/NFAT, or HNF4*α*. Please note that an additional HNF4*α* binding region in the CXCL2 proximal promoter identified via data mining of chromatin immunoprecipitation-sequencing (ChIP-seq) of HNF4 binding in human livers (GSE22078) was left unchanged in the HNF4A mutant vector, and one additional nucleotide substitution was present between the two mutated NF-*κ*B sites in the NF-*κ*B mutant plasmid (see *Supplementary 2* for primers used in mutation promoter sequences). The other mutant reporter vector had deletion of the first upstream 407 base pairs of the promoter sequence, a region which contains multiple glucocorticoid response elements (GREs) and the HNF4 binding site mutated in the HNF4-mutant reporter vector (Figure 9(a)).

Mutation of the HNF4-binding site and 407-base-pair deletion had little or no effect on the basal promoter activity of CXCL2, whereas mutations of either (or both) NF-*κ*B/NFAT- and CRE-binding sites dramatically decreased the basal expression of this promoter in both cell types. In HEPG2 cells, NF-*κ*B mutant was 52% the wildtype (WT) promoter activity, Cre mutant was 36% WT, and NF-*κ*B/Cre double mutant was 19% WT (Figure 9(b)). In HEK293T cells, NF-*κ*B mutant was 19% WT, CRE mutant was 38% WT, and double mutant was 10% WT (Figure 9(c)).

### 3.9. Activation of CXCL2 Promoter by GR in HEPG2 Cells was through a 407-bp Sequence, and Activation by TNF*α* was through the NF-*κ*B/NFAT Binding Sequence

**Figure 10:**
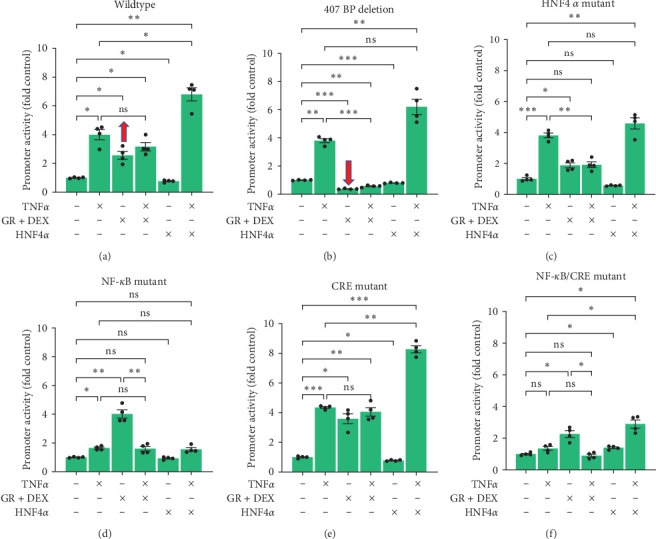
Reporter activities of wildtype and mutant CXCL2 promoters in HEPG2 cells. HEPG2 cells were transfected with luciferase reporter plasmids for (a) wildtype, (b) 407 bp deletion, (c) HNF4*α* mutant, (d) NF-*κ*B mutant, (e) CRE mutant, and (f) NF-*κ*B/CRE dual mutant CXCL2 promoters, dexamethasone groups transfected with GR expression plasmid, HNF4A groups transfected with HNF4*α*1 expression plasmid, and incubated overnight. The following day cells were treated with dexamethasone 10 nM, TNF*α* 20 ng/mL, and/or DMSO control for 24 hr; and then promoter activities were quantified by dual-luciferase assay. Mean ± SE, *n* = 4 replicate wells per sample, and *⁣*^*∗*^*p* ≤ 0.05, *⁣*^*∗*^*⁣*^*∗*^*p* ≤ 0.01, and *⁣*^*∗*^*⁣*^*∗*^*⁣*^*∗*^*p* ≤ 0.001. ns, not significantly different.

Besides just comparing basal expression of these promoters, we also determined the effects of DEX-GR, TNF*α*, and/or HNF4*α* on these mutant promoters. In HEPG2 cells, DEX-GR increased CXCL2 promoter activities 2- to 4-fold for all promoters except the promoter with the 407-base-pair deletion (Figure 10(a)–10(f)). Since, as mentioned above, this region contains six predicted GREs (*Supplementary 2*), these results are expected. When this region of the promoter was missing, DEX-GR inhibited rather than induced CXCL2 promoter activity in HEPG2 cells, decreasing the mutant promoter's activity to 37% (Figure 10(b)). Interestingly, DEX-GR also inhibited TNF induction of CXCL2 promoter when the HNF4-binding site was mutated (Figure 10(c)), which is consistent with the important role of HNF4*α* in determining the DNA-binding and transcriptional activities of GR in hepatocytes [32].

TNF*α*, like DEX, induced CXCL2 promoter activity in HEPG2 cells. WT, 407-base-pair deletion, HNF4A mutant, and CRE mutant promoters all showed a strong 4-fold increase in activity with TNF*α* treatment. The NF-*κ*B mutant showed only a moderate 64% increase in activity by TNF (Figure 10(d)), whereas the NF-*κ*B/CRE double-mutant promoter lost the response to TNF (Figure 10(f)). Since it is well known that TNF*α* is upstream of NF-*κ*B and CRE signaling [33, 34], it is not surprising that mutation of both the NF-*κ*B- and CRE-binding sites resulted in a complete loss of TNF*α* induction of the CXCL2 promoter. In addition, TNF*α* inhibited DEX induction of CXCL2 in both the NF-*κ*B (Figure 10(d)) and NF-*κ*B/CRE double-mutant (Figure 10(f)) promoters, which is likely due to the direct inhibitory effects of TNF*α* on GR [35].

### 3.10. TNF*α* Synergized with HNF4*α* to Induce CXCL2 Promoter Activity in HEPG2 Cells

One interesting observation was that overexpression of HNF4*α* combined with TNF*α* treatment increased CXCL2 promoter activity higher than either alone, demonstrating a synergistic effect between HNF4*α* and TNF*α* in the HEPG2 cells (Figure 10). This was particularly evident in the WT promoter (TNF*α* up 4-fold, HNF4*α* down to 76%, combination up to 7-fold) (Figure 10(a)), the 407-base-pair deletion promoter (TNF*α* up 4-fold, HNF4*α* down to 80%, combination up to 6-fold, *p*=0.09) (Figure 10(b)), and the CRE-mutant promoter (TNF*α* up 4-fold, HNF4*α* down to 78%, combination up to 8-fold) (Figure 10(e)). The synergistic induction of CXCL2 promoter by TNF and HNF4*α* was lost when the NF-*κ*B or HNF4 binding site was mutated (Figures 10(c) and 10(d)) but maintained in the NF-*κ*B/CRE double mutant (TNF*α* and HNF4*α* each up 1.4-fold and combination up to 2.9-fold) (Figure 10(f)).

### 3.11. In HEK293T Cells GR Inhibited CXCL2 in Every Case, and there was No Synergy between HNF4*α* and TNF*α*

**Figure 11:**
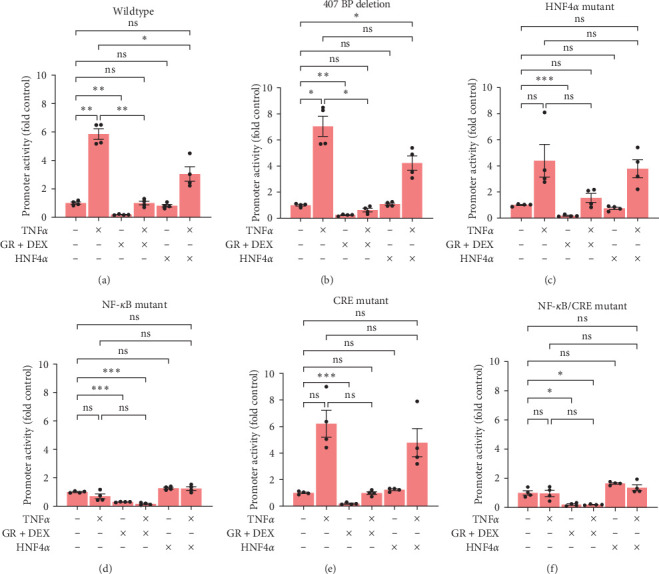
Reporter activities of wildtype and mutant CXCL2 promoters in HEK293T cells. HEK293T cells were transfected with luciferase reporter plasmids for (a) wildtype, (b) 407 bp deletion, (c) HNF4*α* mutant, (d) NF-*κ*B mutant, (e) CRE mutant, and (f) NF-*κ*B/CRE dual mutant CXCL2 promoters, dexamethasone groups transfected with GR expression plasmid, HNF4A groups transfected with HNF4*α*1 expression plasmid, and incubated overnight. The following day cells were treated with dexamethasone 10 nM, TNF*α* 20 ng/mL, and/or DMSO control for 24 hr; and then promoter activities were quantified by dual-luciferase assay. Mean ± SE, *n* = 3–4 replicate wells per sample, and *⁣*^*∗*^*p* ≤ 0.05, *⁣*^*∗*^*⁣*^*∗*^*p* ≤ 0.01, and *⁣*^*∗*^*⁣*^*∗*^*⁣*^*∗*^*p* ≤ 0.001. ns, not significantly different.

Unlike the HEPG2 cells, HEK293T cells showed a decrease in CXCL2 promoter activities with DEX treatment for all the promoters, ranging from 30% control down to 18% control (Figure 11). In fact, the WT promoter acted remarkably similar to the 407-base-pair deletion promoter in HEK293T cells (Figures 11(a) and 11(b)) despite the dramatic difference in these two promoters in the HEPG2 cells. TNF*α* still appeared to be working through the NF-*κ*B-binding site, with complete loss of induction in the NF-*κ*B mutants (Figures 11(d) and 11(f)). However, the synergy seen between TNF*α* and HNF4*α* in the HEPG2 cells was completely nonexistent in the HEK293T cells, with combination values in each case falling somewhere between TNF*α* alone and HNF4*α* alone. We conclude that the CXCL2 promoter was activated and regulated differently in these two cell lines, with GR inducing and combination of TNF*α* and HNF4*α* synergistically inducing CXCL2 promoter activity in HEPG2 cells but not in HEK293T cells.

## 4. Discussion

### 4.1. Overall Logic of Paper

Sepsis is a widespread and life-threatening problem affecting, per the CDC, 1.7 million patients per year in the United States alone, and tens or even hundreds of thousands of patients succumb with or from sepsis each year [36]. Sepsis is a leading cause of death in severe AH [9, 37], and pneumonia and spontaneous bacterial peritonitis are common in severe AH [38, 39]. Since sepsis is an exaggerated inflammatory response to infection, it would intuitively seem that decreasing the inflammatory response (a classical function of GCs) would lead to significant survival benefits. Surprisingly, numerous trials including thousands of patients have shown questionable survival benefit with GC treatment for sepsis except in the most severe cases [40–42], leaving a major gap in treatment options for many of these 1.7 million patients.

This lack of efficacy is bewildering, given the fact that GCs are typically a primary means of limiting an inflammatory response. However, a few factors could be at play in the setting of sepsis. For one thing, it is well known that high doses of GCs are immunosuppressive; thus, utilizing GCs for septic patients is a delicate balance between worsening the root infection and predisposition to repeat infection versus improving the acute hyperinflammatory reaction. For another thing, GC responsiveness is decreased during sepsis [43]. This complication could be attributed to either a decrease in GR expression and/or to loss of responsiveness of the receptor [43]. Perhaps due to these or other undiscovered reasons, GCs have time and again proven to be of limited efficacy in treating sepsis, despite being intuitively the best option for reversing this life-threatening, hyperreactive, hyperinflammatory state that claims the lives of so many people each year.

CXCL1, CXCL2, and CXCL8 critically regulate neutrophil functions. Dysfunction of neutrophils plays a key role in liver injury and increased infection in severe AH. As the most abundant immune cells in humans, neutrophils play multifaceted roles in anti-infection, inflammatory injury, and tissue repair [44, 45]. For example, neutrophils have a dual role in liver injury: neutrophil extracellular traps, degranulation, and oxidative burst cause liver injury, whereas neutrophils can also promote the resolution of inflammation and liver repair by (1) phagocytic clearance of necrotic/apoptotic cells, (2) proresolving of macrophages, (3) regulation of miR-223, and (4) production of hepatocyte growth factor that stimulates liver regeneration [46–49]. CXCL1 and CXCL8 recruit neutrophils to the liver in severe AH [50–52], and human CXCL8 aggravates AH in mice [53, 54]. Infiltration and activation of neutrophils play a key role in AH pathogenesis [55, 56]. By contrast, chronic activation of neutrophils decreases their anti-infection capability in AH [48], which may be the underlying mechanism of increased infections in these patients.

CXCL2 is highly expressed in normal human hepatocytes [20, 57] and is homeostatic or protective. It is markedly down-regulated in AH and liver cancer [7, 58–60], whereas hepatic production and serum CXCL1 and CXCL8 are highly elevated and correlated AH severity in humans [7, 51, 61–65]. Our data mining found that hepatic CXCL2 mRNA expression was also markedly down-regulated in patients with nonalcoholic steatohepatitis (*Supplementary 1*). Hepatocyte death is reduced by 5 ng/mL each of CXCL1 and CXCL2 [66], and CXCL2 promotes liver regeneration and protects against adenovirus- and acetaminophen-induced liver injury [67, 68]. Interestingly, our data mining (GSE151648) found an association of a strong hepatic induction of CXCL2 mRNA (up 2.1-fold) with a lack of reperfusion liver injury in patients after liver transplantation (*Supplementary 1*). In addition, human CXCL2 can synergize with granulocyte colony-stimulating factor to rapidly mobilize the bone marrow early hematopoietic stem and progenitor cells [69, 70], which may promote liver regeneration and enhance the immune function in sepsis. Thus, hepatic CXCL2 deficiency will increase liver injury and the susceptibility to infections.

This study was performed to understand the GR's effect on chemokine and APP expression in the liver. We hypothesized that by increasing GR activity in the liver, chemokine imbalances seen in diseases such as AH [71] could be ameliorated, thus treating or preventing the development of sepsis in this high-risk patient group [9]. To summarize our findings, GR's effect on chemokine expression is more complicated than a simple anti-inflammatory inhibition. The effects are patient specific, cell-type specific, and chemokine specific.

### 4.2. Cell-Type-Specific Regulation of CXCL2

Our data found that DEX inhibited the induction of CXCL2 by LPS in human WB (*Supplementary 1*) and inhibited CXCL2 promoter activity in nonliver HEK293T cells. However, in liver-derived HEPG2 cells, the opposite was true—GR induced CXCL2 promoter activity. In most conditions analyzed in PHH, DEX showed moderate induction or no effect on CXCL2 mRNA expression, cell-associated protein levels of CXCL2, or protein levels of CXCL2 in the media.

To elucidate the mechanism of cell-specific regulation of CXCL2, we analyzed several regions of the CXCL2 promoter and noted that a 407-base-pair sequence with a cluster of predicted GR binding sites is necessary for GR induction of CXCL2 in the HEPG2 cells. When this region was missing, GR's effects were completely opposite, inhibiting CXCL2 promoter activity instead of inducing it in HepG2 cells. However, in HEK293T cells, GR inhibited CXCL2 expression in every promoter, and GR's effect on the 407-base-pair-deletion promoter appeared nearly identical to its effects on the WT promoter. A reasonable hypothesis would be that GR works through the NF-*κ*B binding site to inhibit CXCL2 expression in both cell types, whereas GR works through the predicted GREs to induce CXCL2 expression in HEPG2 cells. However, even this hypothesis would not give a full explanation, as DEX still decreased the activity of CXCL2 promoter with mutation of the NF-*κ*B site in HEK293T cells. Thus, GR must be working through an additional/different region(s) of the promoter, unless the NF-*κ*B mutant promoter retained mild residual NF-*κ*B activity. Different cell types utilizing different promoter regions is not unique to CXCL2 expression. Kumon et al. [72], reported that in HEPG2 cells, an NF-*κ*B binding site in the SAA1 promotor led to synergistic gene induction by DEX and the inflammatory cytokine IL-1, contrasting this with a human aortic smooth muscle cell line where a CCAAT/enhancer-binding protein alpha/beta (CEBP*α*/*β*) binding site in the promoter enabled induction by DEX without IL-1. Further research is needed to elucidate more precise promoter region(s) or cofactors involved in cell-specific regulation of CXCL2 by the GR.

Several other variables could also be responsible for the differential responses of CXCL2 promoter to DEX treatment in the PHH and cell lines. For one thing, in the HEPG2 and HEK293T cell lines, GR was overexpressed via transfection with a GR expression vector. This could explain the stronger effects, both positive and negative, seen following DEX treatment in the tumor cell lines as opposed to the primary cells, which relied on endogenous GR only. In addition, HEPG2 cells have vastly different drug metabolism profiles than do primary hepatocytes, despite both being liver derived [73]. Thus, even if drug dosage is identical, drug response will likely vary greatly between these cells. Drug dosage varied, however, in our experiments from 10 nM in the HEPG2 and HEK293T cell lines to 100 nM or even 1,000 nM in the PHH. Although it seems somewhat counterintuitive that higher drug dosage would have less effects than lower drug dosage, this is possible and, in fact, we observe this in the PHH qPCR data for CXCL8 (Figure 1(c)). Another variable to consider is the presence of FBS in the tumor cells' culture media but serum-free media as the PHH culture media. It is known that FBS can affect chemokine expression [74]; thus serum-free media versus serum-supplemented media may play a role in the differences in GR regulation of CXCL2 between PHH and cell lines.

### 4.3. HNF4*α* Effects in Early versus Late-Stage Liver Disease

In the same dual luciferase assay, we also noted a synergy between HNF4*α* and TNF*α* in the HEPG2 cells, inducing CXCL2 promoter activity. In the HEK293T cells, however, HNF4*α* seemed to counteract TNF*α*, as combined treatment led to promoter activation somewhere between the individual treatments.

Interestingly, patients with AH have a deficiency of both HNF4A [75] as well as a decreased level of CXCL2 [7]. Previous work in this lab showed a direct correlation between murine GR expression and HNF4*α* expression, demonstrating that loss of GR led to a loss of HNF4*α* as well [76]. Moreover, GR and HNF4*α* coordinately regulate hepatic gene expression [32, 76]. We can hypothesize that in healthy humans, high levels of GR and HNF4*α* lead to high hepatic basal expression of CXCL2 and induction of CXCL2 in early inflammatory liver diseases, whereas patients with late-stage hepatic diseases have a deficiency of HNF4*α* and GR, leading to the decreased CXCL2 levels observed in patients with AH, nonalcoholic steatohepatitis, and liver cancer. HNF4*α* deficiency is a driver of hepatocellular failure in AH [75]. Thus, restoring or increasing GR activity in the liver would not only increase CXCL2 expression through GR itself but also through improved HNF4*α* levels as well.

### 4.4. Differences in Chemokine Regulation (CXCL1, CXCL2, and CXCL8)

In primary hepatocytes from one donor, DEX treatment inhibited CXCL1 and CXCL8 protein expression and secretion induced by TNF*α* or LPS, whereas CXCL2 upregulation was largely unaffected by DEX treatment. In the other patient, GR strongly inhibited LPS-induced CXCL1 and CXCL8 translation and transcription, while CXCL2 transcription tended to increase with DEX treatment, although secreted CXCL2 induced by LPS dropped slightly with DEX.

It has been recognized for several decades that elevated serum CXCL8 (IL-8) is closely linked to liver injury in AH patients [77]. Dominguez et al. [7] investigated the correlation between several chemokines (including CXCL1–8 and CXCL10) and mortality in patients with AH. Interestingly, although CXCL1 mRNA was extremely elevated in AH patients (over 700 times control levels), it did not associate with mortality in these patients. They reported that CXCL8 mRNA and hepatic protein (but not serum protein) did, however, corelate with mortality. (As an interesting and likely relevant side note, it is well known that the chemokine receptor CXCR1 is selective for the CXCL8 ligand; whereas the CXCR2 chemokine receptor can be activated by several ligands including CXCL1, CXCL2, CXCL8, and others [78, 79]). The 90-day mortality of AH was over 20%, and nearly a quarter of these deaths were due to sepsis. Forty-four percent of AH patients suffered bacterial infection during their hospital stay. They suggested that drugs regulating chemokines may be a beneficial treatment for AH patients. Systemic GCs are often prescribed for AH patients, but the benefits of this treatment can be difficult to interpret [80]. In our study, GCs were able to limit CXCL8 mRNA, protein in cell lysates, as well as protein secretion into culture media, supporting our hypothesis that GCs could be protective in AH and sepsis if nonhepatic side effects can be limited.

Of particular interest is the observation that secretion profiles differ markedly between chemokines, for example, between CXCL1 and CXCL2. The fact that CXCL2 has high basal expression and much higher cell-associated-versus-secreted profile relative to CXCL1 and CXCL8 suggests a homeostatic role of CXCL2 in human livers. CXCL1 and CXCL2 have very similar *N*-terminal signal peptides suggesting both are secreted through the endoplasmic reticulum (see the chapter titled “The Endoplasmic Reticulum” [81]). Alternatively, this could be due to differences in chemokines binding to the extracellular matrix and/or receptor [82]. Although we compared cellular lysates versus media, we did not analyze whether the differences we observed were due to distinctions in intracellular retention or to extracellular glycosaminoglycan binding of the chemokines. It is reasonable to assume that chemokines trapped in the extracellular matrix were classified as “cell-associated” by our experimental design as opposed to “secreted” into the media. A paper by Baumann et al. [83] published in 1983 made a fascinating discovery that mouse primary hepatocytes treated with DEX plus proinflammatory media (from activated monocytes) had less sialylation of glycoproteins than hepatocytes treated with just the proinflammatory media. Since chemokines bind glycosaminoglycans [84], we could hypothesize that changes in glycoprotein sialylation due to DEX treatment could affect chemokine retention in the extracellular matrix versus secretion to the media [85]. Thus, it is unclear from our research whether intracellular retention or glycosaminoglycan binding is responsible for the differing chemokine secretion profiles. Further research would be required to answer this question.

Although in our research we focused on hepatocyte-like HEPG2 cells and PHH, a recent paper in Nature Communications studying the upregulation of various chemokines in response to TNF*α* and AH reported that liver sinusoidal endothelial cells were a greater contributor than hepatocytes of CXCL1, CXCL6, and CXCL8 but that CXCL2 expression was greatest in hepatocytes [20]. Although HepG2 cells, but not PHH, was used in that comparative study [20], an appropriate followup to both our study as well as theirs would be to study the GR's effects on chemokine expression in liver sinusoidal endothelial cells. Our data showing that TNF*α* works through the NF-*κ*B binding site in the CXCL2 promoter agrees with their data for TNF*α* and NF-*κ*B regulation of CXCL1, CXCL6, and CXCL8. However, both their data and ours show a difference in the overall regulation of CXCL1 versus CXCL2.

### 4.5. Differential Effects of GR in the Setting of TNF*α* versus LPS

In many cases, GR will either augment or inhibit both the effects of TNF*α* and LPS. Occasionally, however, as in the case of the CXCL10 in primary hepatocytes, GR will augment one and inhibit the other. In our experiments, DEX typically affected LPS effects much more than it did TNF*α* effects. Since TNF*α* is an endogenous protein but LPS is a bacterially derived endotoxin, LPS treatment models a much more severe infectious/inflammatory disease than TNF*α* treatment does. In the early stage of bacterial infection, the mobilization and recruitment of neutrophils by inflammatory chemokines, stimulated by TNF*α*, plays an important role in anti-infection. By contrast, the hyperinflammation caused by LPS in the severe/late stage of bacterial infection is a major driver of sepsis. Thus, it makes sense that DEX's inhibitory effects on inflammatory chemokines would be more important in a severe infection than they would be in a less severe setting. In the early infection, specifically activating hepatic GR will be safer to avoid immunosuppression caused by systemic exposure to GCs.

### 4.6. TNF-GR Differences on CXCL10 in PHH versus HEPG2, and Differential Effects of GC-GR on TNF- and LPS-Induction of CXCL10 in PHH

What is intriguing is the difference in DEX effects on CXCL10 expression in primary hepatocytes versus HEPG2 cells. In primary hepatocytes, DEX had no effect or even augmented TNF*α* induction of CXCL10 (Figures 3(a) and 3(d)), but in HEPG2 cells DEX counteracted it (Figure 8(d)). This could be dose-related, as DEX 100 or 1,000 nM and TNF*α* 50 ng/mL were used on the primary hepatocytes but only DEX 10 nM (with transfection of additional GR) and TNF*α* 20 ng/mL was used in the HEPG2 cells. Alternatively, it could represent a subtle regulatory difference between cell types like that seen with CXCL2 expression.

GC has been shown to inhibit the induction of CXCL10 in tubular epithelial cells to prevent renal infiltration of CXCR3+CD4+ T cells and subsequent renal tissue damage in patents and mice with crescentic glomerulonephritis [86]. CXCL10 is different from the first three CXC chemokines in that it has a different receptor, CXCR3, than the other three, which target CXCR1 and/or CXCR2. CXCL10 also interacts with T cells, whereas the other three signal neutrophils [3]. T cells have a dual role in sepsis: In severe sepsis models, activated T cells can increase sepsis morbidity and tissue injury; conversely, in less severe models, functional T cells decrease mortality and bacterial load [87]. In parallel, CXCL10 protects moderate sepsis but aggravates tissue injury and mortality in severe septic shock induced by cecal ligation and puncture [88–90]. Therefore, GC's potentiation of TNF-induction of CXCL10 in early/moderate sepsis and attenuation of LPS-induction of CXCL10 in hepatocytes during severe sepsis suggest that specifically activating hepatocellular GR to differentially modulate TNF- and LPS-induction of CXCL10 will be beneficial for both moderate and severe sepsis.

A paper by Zhang et al. [91], described elevated serum CXCL10 in patients with fatty liver and nonalcoholic steatohepatitis, noting histological correlation between CXCL10 levels and liver disease. They demonstrated that in a mouse model of steatohepatitis, CXCL10 was upstream of TNF*α* expression and NF-*κ*B activity and that genetic knockout or antibody inhibition of CXCL10 significantly limited the development of steatohepatitis. CXCL10 can either aggravate or inhibit viral infections [92]. Since patients with fatty liver are more likely to develop bacterial infections or sepsis [93], limiting overexpression of CXCL10 could be protective against worsening liver disease, bacterial infection, and even sepsis, although this might require long-term prophylaxis rather than short-term treatment of acute disease.

### 4.7. Regulation of APPs by GC-GR

Some APPs were affected by DEX treatment in PHH besides the chemokines mentioned above. SAA1 improves neutrophil and monocyte viability [94, 95] and is chemoattractant for neutrophils, mononuclear cells, and other white blood cells [94, 96, 97]. It also synergizes with CXCL8 to recruit white blood cells [94, 97, 98]. Although there are reports of SAA1 inducing secretion of matrix metalloproteinases [99], regulating reactive oxygen species [100, 101], and upregulating chemokines [97], there is also evidence that these effects may in some cases be artifact due to bacterial contamination in recombinant SAA1 products instead of due to SAA1 itself [94]. Alternatively, the inflammatory effects of SAA1 may be related to whether SAA1 is bound to or released from high-density lipoproteins [102]. Perhaps most importantly, SAA1 binds LPS, improving macrophage phagocytosis of this bacterial toxin [103], and SAA binds to many Gram-negative bacteria including *E. coli* and *Pseudomonas aeruginosa* to enhance their phagocytosis and anti-infectious immune response [104]. Higher SAA in humans is associated with less severe chronic liver diseases [105], and SAA antibody worsens liver fibrosis [106]. Sepsis nonsurvivors have lower serum SAA than survivors [107]; and SAA1 supplementation improves, whereas SAA inhibition worsens polymicrobial sepsis in mice [108, 109]. Thus, SAA is critical in anti-infection, tissue repair, and anti-fibrosis during sepsis. Our novel data in PHH agree with published literature in hepatoma cells, which document a synergy between DEX and various proinflammatory cytokines and LPS in inducing dramatic upregulation of SAA1 [110]. Liver-specific activation of GR and the resultant synergistic induction of SAA1 will be beneficial for inflammatory liver diseases and sepsis.

Of particular interest are the regulation of CD163 and HAMP by GC-GR. Per single-cell sequencing [111] (Human Protein Atlas, https://www.proteinatlas.org, version 22.0), CD163 is expressed highly in liver-specific Kupffer cells or other macrophages and moderately in human hepatocytes (CD163 data available from https://www.proteinatlas.org/ENSG00000177575-CD163/single%2Bcell%2Btype/liver). HAMP is expressed primarily by hepatocytes (HAMP data available from https://www.proteinatlas.org/ENSG00000105697-HAMP/single%2Bcell%2Btype/liver). Both these genes are involved in reducing iron levels in the blood during infection [24, 112], which is important to prevent ferroptosis and bacteria overgrowth [25, 26]. However, septic patients also have an increased risk of anemia including iron-deficiency anemia [113]. CD163 is an endocytic receptor essential for the removal of the hemoglobin–haptoglobin complex formed during hemolysis [114]. HAMP, on the other hand, regulates iron absorption from the gut and iron released from the liver or macrophages into the bloodstream [115]. DEX treatment counteracted TNF*α* and LPS downregulation of CD163 and even increased expression above baseline for both treatments, which will be beneficial for both moderate and severe sepsis. By contrast, on HAMP, DEX had no effect when combined with TNF*α* but dramatically increased expression in the setting of LPS (i.e., bacterial infection). Because elevation of TNF*α* also occurs in sterile inflammation, elevation of HAMP will increase the risk of iron-deficiency anemia. Thus, in hepatocytes DEX induced an antibacterial response to LPS exposure by upregulating iron-reducing genes CD163 and HAMP.

## 5. Conclusion

In conclusion, GR regulation of chemokines in the liver is complex, showing patient-specific, stimulus (TNF/LPS)-specific, dose-specific, and chemokine-specific effects. However, the overall GR trend seems to be restorative in liver cells, selectively limiting the inflammatory overexpression of CXCL1 and CXCL8 while simultaneously sparing CXCL2, which is downregulated in various liver diseases but may be crucial for hepatic health. CXCL2 appears to be a homeostatic chemokine, with high basal levels and a localization predominantly cellular or cell-associated. Downregulation of hepatic CXCL2, correlated with liver damage, is likely due in part to the deficiencies of GR and HNF4*α* often seen in chronic liver diseases. In contrast to the PHH and HEPG2 cells, in human WB and a nonliver cell line GR decreased both CXCL2 and CXCL8. We traced this divergence of GR activity to a 407-base pair region of the CXCL2 promoter and an HNF4-binding site within this region, whose losses resulted in GR-induced decreases of basal and TNF-induced activities of the CXCL2 promoter, respectively. Our hypothesis is that specifically stimulating hepatic (as opposed to systemic) GR activity in AH patients with dysregulated expression of chemokines and APPs may be more beneficial in treating AH and preventing/treating sepsis in part by restoring a more appropriate chemokine balance and inducing APPs. In the early/moderate stage of infection, activation of hepatocellular GR will induce CXCL2 and APPs such as SAA1 and moderately inhibit the TNF-stimulated expression/secretion of most inflammatory cytokines/chemokines, with the exception of CXCL10, which can directly kill both Gram-positive and Gram-negative bacterial pathogens in vitro [116]. In the more severe stage of bacterial infection associated with markedly elevated LPS, activation of hepatocellular GR will cause more marked induction of APPs SAA1 and HAMP and inhibition of inflammatory cytokines/chemokines, including CXCL10, to help fight bacterial infections and protect against tissue damage caused by hyperinflammation.

## Data Availability

All data are provided in this manuscript or accessible via GEO Datasets.

## Additional Points

*Limitations*. As mentioned above, cytokine levels in media were measured in terms of picograms per milliliter, whereas the cytokine levels in the cellular lysate were measured in terms of picograms per milligram protein. This was due to a failed attempt to divide the PHH cells from Donor 2 for use in both qPCR and ELISA assays. Because of this, we were unable to combine the results of media and lysates to show what fraction was secreted versus what fraction was cell-associated for each CXC chemokine, and instead we used the ratios between different CXC chemokines shown above. It would be interesting in future studies to show percentages or fractions of the whole. In a preliminary approximation using media and lysate from patient 1 (*Supplementary 1*), the majority of total CXCL2 proteins were associated with PHH, whereas the majority of CXCL1 and CXCL8 proteins were secreted out of PHH. Another limitation is the fact that in the cell lysate experiments we normalized each sample to protein concentration in the lysate, whereas the media experiments were based on volume and were not normalized. This raises the alternate hypothesis that total secreted protein levels rather than specific cytokine levels may have been affected by treatments, for example, if cell viability was affected. While this is a definite possibility, the fact that secretion profiles differ largely among these chemokines makes this less likely. It has been recognized for approximately 40 years that DEX in cell culture media stabilizes the differentiation of dissociated primary hepatocytes [83], although even 100 nM DEX in the culture media does not prevent proteomic changes due to dedifferentiation within even 24 hr [117]. This is something of a limitation in our study, as DEX was the primary treatment being analyzed. Upon receipt of the PHH, we changed the media to DEX-free and serum-free media overnight and the following day added DEX for 24 hr in select wells as specified above. Unfortunately, limitations such as dedifferentiation are nearly inevitable in dissociated hepatocyte monolayers. It would be useful to follow up our experiments in chimeric/humanized mice or mouse-passaged PHHs to minimize the potential nonspecific effects due to dedifferentiation innate to dissociated hepatocyte culture [118].

*Perspective*. Increased risk of serious infections by systemic GC treatment is a major side effect of GCs that may offset their benefit in AH [119–123]. Conversely, hepatic GR deficiency worsened liver failure and mortality in sepsis [124]. GC is avoided in AH patients with infections [71]. To improve the current GC therapy of severe AH, it is imperative to understand how GC regulates cell-specific CXC chemokine expression, neutrophil function, disease severity, and infection in AH. Pioneer and liver-enriched factors, particularly forkhead Box A2 (FOXA2), mediate liver-specific DNA binding and transactivation of GR [125]; however, our preliminary data (not shown) showed that combination of FOXA2, HNF4*α*, and GR still failed to activate the CXCL2 promoter in HEK293 cells, suggesting that other liver-enriched factor(s) may be needed for hepatocyte-specific activation of CXCL2 by GR. Little is currently known about how GCs regulate CXC chemokines and the differential roles of these CXC chemokines in liver injury and infection in AH. Bridging this key knowledge gap will improve the current GC therapy and decrease the risk of sepsis in AH patients. Currently, the molecular mechanism and functional significance of hepatic dramatic down-regulation of CXCL2 but induction of CXCL8 remain unknown. There is no report on changes in circulating CXCL2 in AH patients. Our preliminary data suggest that the majority of CXCL2 proteins produced by normal human hepatocytes are cell-associated rather than secreted into the media, and thus the substantial hepatic down-regulation of CXCL2 in AH patients may not be reflected by marked changes in blood levels of CXCL2. It will be interesting to determine whether the serum ratio of CXCL8/CXCL2 would be a good biomarker for AH severity and GC response in AH patients. Further research is needed to answer these questions.

## Supplementary Materials

Supplementary 1 Figure S1: effects of glucocorticoid receptor (GR) on the reporter activities of FKBP5 intron5–6 in HepG2 cells and HEK293 cells. Figure S2: mRNA expression in healthy versus NASH livers. Figure S3: CXCL2 mRNA pre- and post-liver transplant. Figure S4: cytokine mRNA changes in human whole blood with LPS and DEX. Figure S5: approximation of chemokine lysate/media percentages.

Supplementary 2 Table S1: primers. Table S2: GRE sites predicted in the human CXCL2 promoter.

## References

[1] Chousterman B. G., Swirski F. K., Weber G. F. (2017). Cytokine storm and sepsis disease pathogenesis. *Seminars in Immunopathology*.

[2] Karin N., Wildbaum G. (2015). The role of chemokines in shaping the balance between CD4^+^ T cell subsets and its therapeutic implications in autoimmune and cancer diseases. *Frontiers in Immunology*.

[3] Saiman Y., Friedman S. L. (2012). The role of chemokines in acute liver injury. *Frontiers in Physiology*.

[4] Strnad P., Tacke F., Koch A., Trautwein C. (2017). Liver—guardian, modifier and target of sepsis. *Nature Reviews Gastroenterology & Hepatology*.

[5] McDonald B., Urrutia R., Yipp B. G., Jenne C. N., Kubes P. (2012). Intravascular neutrophil extracellular traps capture bacteria from the bloodstream during sepsis. *Cell Host & Microbe*.

[6] Jain S., Gautam V., Naseem S. (2011). Acute-phase proteins: as diagnostic tool. *Journal of Pharmacy and Bioallied Sciences*.

[7] Dominguez M., Miquel R., Colmenero J. (2009). Hepatic expression of CXC chemokines predicts portal hypertension and survival in patients with alcoholic hepatitis. *Gastroenterology*.

[8] Yan X., Liang J., Li X. (2023). Identify key genes correlated to ischemia-reperfusion injury in aging livers. *Disease Markers*.

[9] Gustot T., Fernandez J., Szabo G. (2017). Sepsis in alcohol-related liver disease. *Journal of Hepatology*.

[10] Alvaro-Meca A., Maté-Cano I., Ryan P., Briz Vónica, Resino S. (2020). Epidemiological trend of sepsis in patients with hospital admissions related to hepatitis C in spain (2000–2015): a nationwide study. *Journal of Clinical Medicine*.

[11] Kempe K. C., Isom H. C., Greene F. E. (1995). Responsiveness of an SV40-immortalized hepatocyte cell line to growth hormone. *Biochemical Pharmacology*.

[12] Gebert C. A., Park S.-H., Waxman D. J. (1997). Regulation of signal transducer and activator of transcription (STAT) 5b activation by the temporal pattern of growth hormone stimulation. *Molecular Endocrinology*.

[13] Zhang Q., Wang J., Deng F. (2015). TqPCR: a touchdown qPCR assay with significantly improved detection sensitivity and amplification efficiency of SYBR green qPCR. *PLoS One*.

[14] Coulibaly A., Velásquez S. Y., Sticht C. (2019). AKIRIN1: a potential new reference gene in human natural killer cells and granulocytes in sepsis. *International Journal of Molecular Sciences*.

[15] Farré D., Roset R., Huerta M. (2003). Identification of patterns in biological sequences at the ALGGEN server: PROMO and MALGEN. *Nucleic Acids Research*.

[16] Messeguer X., Escudero R., Farré D., Núñez O., Martínez J., Albà M. M. (2002). PROMO: detection of known transcription regulatory elements using species-tailored searches. *Bioinformatics*.

[17] Guo S., Lu H. (2019). Novel mechanisms of regulation of the expression and transcriptional activity of hepatocyte nuclear factor 4*α*. *Journal of Cellular Biochemistry*.

[18] Parameswaran N., Patial S. (2010). Tumor necrosis factor-*α* signaling in macrophages. *Critical Reviews™ in Eukaryotic Gene Expression*.

[19] Spencer N. Y., Zhou W., Li Q. (2013). Hepatocytes produce TNF-*α* following hypoxia-reoxygenation and liver ischemia-reperfusion in a NADPH oxidase- and c-Src-dependent manner. *American Journal of Physiology-Gastrointestinal and Liver Physiology*.

[20] Liu M., Cao S., He L. (2021). Super enhancer regulation of cytokine-induced chemokine production in alcoholic hepatitis. *Nature Communications*.

[21] Yao Z., Mates J. M., Cheplowitz A. M. (2016). Blood-borne lipopolysaccharide is rapidly eliminated by liver sinusoidal endothelial cells via high-density lipoprotein. *The Journal of Immunology*.

[22] Pu Z., Bao X., Xia S., Shao P., Xu Y. (2022). Serpine1 regulates peripheral neutrophil recruitment and acts as potential target in ischemic stroke. *Journal of Inflammation Research*.

[23] Levine J. A., Oleaga C., Eren M. (2021). Role of PAI-1 in hepatic steatosis and dyslipidemia. *Scientific Reports*.

[24] Nemeth E., Ganz T. (2009). The role of hepcidin in iron metabolism. *Acta Haematologica*.

[25] Cross J. H., Bradbury R. S., Fulford A. J. (2015). Oral iron acutely elevates bacterial growth in human serum. *Scientific Reports*.

[26] Chen J., Li X., Ge C., Min J., Wang F. (2022). The multifaceted role of ferroptosis in liver disease. *Cell Death and Differentiation*.

[27] de Waal G. M., de Villiers W. J. S., Forgan T., Roberts T., Pretorius E. (2020). Colorectal cancer is associated with increased circulating lipopolysaccharide, inflammation and hypercoagulability. *Scientific Reports*.

[28] Hubler T. R., Scammell J. G. (2004). Intronic hormone response elements mediate regulation of FKBP5 by progestins and glucocorticoids. *Cell Stress & Chaperones*.

[29] Robinson J. T., Thorvaldsdóttir H., Winckler W. (2011). Integrative genomics viewer. *Nature Biotechnology*.

[30] Thorvaldsdóttir H., Robinson J. T., Mesirov J. P. (2013). Integrative genomics viewer (IGV): high-performance genomics data visualization and exploration. *Briefings in Bioinformatics*.

[31] Lu H. (2016). Crosstalk of HNF4*α* with extracellular and intracellular signaling pathways in the regulation of hepatic metabolism of drugs and lipids. *Acta Pharmaceutica Sinica B*.

[32] Hunter A. L., Poolman T. M., Kim D. (2022). HNF4A modulates glucocorticoid action in the liver. *Cell Reports*.

[33] Hayden M. S., Ghosh S. (2014). Regulation of NF-*κ*B by TNF family cytokines. *Seminars in Immunology*.

[34] Gustin J. A., Pincheira R., Mayo L. D. (2004). Tumor necrosis factor activates CRE-binding protein through a p38 MAPK/MSK1 signaling pathway in endothelial cells. *American Journal of Physiology-Cell Physiology*.

[35] Dendoncker K., Timmermans S., Vandewalle J. (2019). TNF-*α* inhibits glucocorticoid receptor-induced gene expression by reshaping the GR nuclear cofactor profile. *Proceedings of the National Academy of Sciences of the United States of America*.

[36] Centers for Disease Control and Prevention, N.C.f.E.a.Z.I.D.N., Division of Healthcare Quality Promotion (DHQP) (2022). What is Sepsis?. https://www.cdc.gov/sepsis/what-is-sepsis.html.

[37] Pérez-Hernández O., González-Reimers E., Quintero-Platt G. (2017). Malondialdehyde as a prognostic factor in alcoholic hepatitis. *Alcohol and Alcoholism*.

[38] Niu B., Kim B., Limketkai B. N. (2018). Mortality from spontaneous bacterial peritonitis among hospitalized patients in the USA. *Digestive Diseases and Sciences*.

[39] Lee D. U., Fan G. H., Hastie D. J. (2021). The impact of malnutrition on the hospital and infectious outcomes of patients admitted with alcoholic hepatitis: 2011 to 2017 analysis of US hospitals. *Journal of Clinical Gastroenterology*.

[40] Liang H., Song H., Zhai R. (2021). Corticosteroids for treating sepsis in adult patients: a systematic review and meta-analysis. *Frontiers in Immunology*.

[41] Rochwerg B., Oczkowski S. J., Siemieniuk R. A. C. (2018). Corticosteroids in sepsis: an updated systematic review and meta-analysis. *Critical Care Medicine*.

[42] Fang F., Zhang Y., Tang J. (2019). Association of corticosteroid treatment with outcomes in adult patients with sepsis: a systematic review and meta-analysis. *JAMA Internal Medicine*.

[43] Vandewalle J., Timmermans S., Paakinaho V. (2021). Combined glucocorticoid resistance and hyperlactatemia contributes to lethal shock in sepsis. *Cell Metabolism*.

[44] Wang J. (2018). Neutrophils in tissue injury and repair. *Cell and Tissue Research*.

[45] Peiseler M., Kubes P. (2019). More friend than foe: the emerging role of neutrophils in tissue repair. *Journal of Clinical Investigation*.

[46] Calvente C. J., Tameda M., Johnson C. D. (2019). Neutrophils contribute to spontaneous resolution of liver inflammation and fibrosis via microRNA-223. *Journal of Clinical Investigation*.

[47] He Y., Rodrigues R. M., Wang X. (2021). Neutrophil-to-hepatocyte communication via LDLR-dependent miR-223-enriched extracellular vesicle transfer ameliorates nonalcoholic steatohepatitis. *Journal of Clinical Investigation*.

[48] Liu K., Wang F.-S., Xu R. (2021). Neutrophils in liver diseases: pathogenesis and therapeutic targets. *Cellular & Molecular Immunology*.

[49] Mayadas T. N., Cullere X., Lowell C. A. (2014). The multifaceted functions of neutrophils. *Annual Review of Pathology: Mechanisms of Disease*.

[50] Takeuchi M., Vidigal P. T., Guerra M. T. (2021). Neutrophils interact with cholangiocytes to cause cholestatic changes in alcoholic hepatitis. *Gut*.

[51] Maltby J., Wright S., Bird G., Sheron N. (1996). Chemokine levels in human liver homogenates: associations between GRO alpha and histopathological evidence of alcoholic hepatitis. *Hepatology*.

[52] Gao B., Ahmad M. F., Nagy L. E., Tsukamoto H. (2019). Inflammatory pathways in alcoholic steatohepatitis. *Journal of Hepatology*.

[53] Chang B., Xu M.-J., Zhou Z. (2015). Short- or long-term high-fat diet feeding plus acute ethanol binge synergistically induce acute liver injury in mice: an important role for CXCL1. *Hepatology*.

[54] Wang Z., Li B., Jiang H. (2021). IL-8 exacerbates alcohol-induced fatty liver disease via the Akt/HIF-1*α* pathway in human IL-8-expressing mice. *Cytokine*.

[55] Bertola A., Park O., Gao B. (2013). Chronic plus binge ethanol feeding synergistically induces neutrophil infiltration and liver injury in mice: a critical role for E-selectin. *Hepatology*.

[56] Ma J., Guillot A., Yang Z. (2022). Distinct histopathological phenotypes of severe alcoholic hepatitis suggest different mechanisms driving liver injury and failure. *Journal of Clinical Investigation*.

[57] Fagerberg L., Hallström B. M., Oksvold P. (2014). Analysis of the human tissue-specific expression by genome-wide integration of transcriptomics and antibody-based proteomics. *Molecular & Cellular Proteomics*.

[58] Lin T., Zhang E., Mai P.-P., Zhang Y.-Z., Chen X., Peng L.-S. (2021). CXCL2/10/12/14 are prognostic biomarkers and correlated with immune infiltration in hepatocellular carcinoma. *Bioscience Reports*.

[59] Ding J., Xu K., Zhang J. (2018). Overexpression of CXCL2 inhibits cell proliferation and promotes apoptosis in hepatocellular carcinoma. *BMB Reports*.

[60] Hoggatt J., Singh P., Tate T. A. (2018). Rapid mobilization reveals a highly engraftable hematopoietic stem cell. *Cell*.

[61] French S. W., Mendoza A. S., Afifiyan N., Tillman B., Vitocruz E., French B. A. (2017). The role of the IL-8 signaling pathway in the infiltration of granulocytes into the livers of patients with alcoholic hepatitis. *Experimental and Molecular Pathology*.

[62] Liu H., French B. A., Nelson T. J., Li J., Tillman B., French S. W. (2015). IL-8 signaling is up-regulated in alcoholic hepatitis and DDC fed mice with Mallory Denk Bodies (MDBs) present. *Experimental and Molecular Pathology*.

[63] Patel O. P., Noor M. T., Kumar R., Thakur B. S. (2015). Serum interleukin 8 and 12 levels predict severity and mortality in patients with alcoholic hepatitis. *Indian Journal of Gastroenterology*.

[64] Huang Y. S., Chan C. Y., Wu J. C., Pai C. H., Chao Y., Lee S. D. (1996). Serum levels of interleukin-8 in alcoholic liver disease: relationship with disease stage, biochemical parameters and survival. *Journal of Hepatology*.

[65] Hill D. B., Marsano L. S., McClain C. J. (1993). Increased plasma interleukin-8 concentrations in alcoholic hepatitis. *Hepatology*.

[66] Kuboki S., Shin T., Huber N. (2008). Hepatocyte signaling through CXC chemokine receptor-2 is detrimental to liver recovery after ischemia/reperfusion in mice. *Hepatology*.

[67] Ren X., Carpenter A., Hogaboam C., Colletti L. (2003). Mitogenic properties of endogenous and pharmacological doses of macrophage inflammatory protein-2 after 70% hepatectomy in the mouse. *American Journal of Pathology*.

[68] Hogaboam C. M., Simpson K. J., Chensue S. W. (1999). Macrophage inflammatory protein-2 gene therapy attenuates adenovirus- and acetaminophen-mediated hepatic injury. *Gene Therapy*.

[69] Fukuda S., Bian H., King A. G., Pelus L. M. (2007). The chemokine GRO*β* mobilizes early hematopoietic stem cells characterized by enhanced homing and engraftment. *Blood*.

[70] Pelus L. M., Bian H., King A. G., Fukuda S. (2004). Neutrophil-derived MMP-9 mediates synergistic mobilization of hematopoietic stem and progenitor cells by the combination of G-CSF and the chemokines GRO*β*/CXCL2 and GRO*β*T /CXCL2*Δ*4. *Blood*.

[71] Lu H. (2022). Narrative review: glucocorticoids in alcoholic hepatitis-benefits, side effects, and mechanisms. *Journal of Xenobiotics*.

[72] Kumon Y., Suehiro T., Faulkes D. J. (2002). Transcriptional regulation of serum amyloid A1 gene expression in human aortic smooth muscle cells involves CCAAT/enhancer binding proteins (C/EBP) and is distinct from HepG2 cells. *Scandinavian Journal of Immunology*.

[73] Guo L., Dial S., Shi L. (2011). Similarities and differences in the expression of drug-metabolizing enzymes between human hepatic cell lines and primary human hepatocytes. *Drug Metabolism and Disposition*.

[74] Fernández-Bertolín L., Mullol J., Fuentes-Prado M. (2015). Effect of lipopolysaccharide on glucocorticoid receptor function in control nasal mucosa fibroblasts and in fibroblasts from patients with chronic rhinosinusitis with nasal polyps and asthma. *PLoS One*.

[75] Argemi J., Latasa M. U., Atkinson S. R. (2019). Defective HNF4alpha-dependent gene expression as a driver of hepatocellular failure in alcoholic hepatitis. *Nature Communications*.

[76] Lu H., Lei X., Winkler R. (2022). Crosstalk of hepatocyte nuclear factor 4a and glucocorticoid receptor in the regulation of lipid metabolism in mice fed a high-fat-high-sugar diet. *Lipids in Health and Disease*.

[77] Ishii K., Furudera S., Kumashiro R. (1994). Role of serum interleukin-8 and intercellular adhesion molecule-1 in the severity of alcoholic hepatitis. *Alcohol and Alcoholism (Oxford, Oxfordshire). Supplement*.

[78] Rajagopalan L., Rajarathnam K. (2004). Ligand selectivity and affinity of chemokine receptor CXCR1. Role of N-terminal domain. *Journal of Biological Chemistry*.

[79] Ahuja S. K., Murphy P. M. (1996). The CXC chemokines growth-regulated oncogene (GRO) *α*, GRO*β*, GRO*γ*, neutrophil-activating peptide-2, and epithelial cell-derived neutrophil-activating peptide-78 are potent agonists for the type B, but not the type A, human interleukin-8 receptor. *Journal of Biological Chemistry*.

[80] Pavlov C. S., Varganova D. L., Casazza G. (2017). Glucocorticosteroids for people with alcoholic hepatitis. *Cochrane Database of Systematic Reviews*.

[81] Cooper G. (2000). *The Cell: A Molecular Approach*.

[82] Sawant K. V., Sepuru K. M., Lowry E. (2021). Neutrophil recruitment by chemokines Cxcl1/KC and Cxcl2/MIP2: role of Cxcr2 activation and glycosaminoglycan interactions. *Journal of Leukocyte Biology*.

[83] Baumann H., Jahreis G. P., Gaines K. C. (1983). Synthesis and regulation of acute phase plasma proteins in primary cultures of mouse hepatocytes. *Journal of Cell Biology*.

[84] Crijns H., Vanheule V., Proost P. (2020). Targeting chemokine–glycosaminoglycan interactions to inhibit inflammation. *Frontiers in Immunology*.

[85] Bryant N. A., Davis-Poynter N., Vanderplasschen A., Alcami A. (2003). Glycoprotein G isoforms from some alphaherpesviruses function as broad-spectrum chemokine binding proteins. *The EMBO Journal*.

[86] Riedel J.-H., Robben L., Paust H.-J. (2023). Glucocorticoids target the CXCL9/CXCL10-CXCR3 axis and confer protection against immune-mediated kidney injury. *JCI Insight*.

[87] Kasten K. R., Tschöp J., Adediran S. G., Hildeman D. A., Caldwell C. C. (2010). T cells are potent early mediators of the host response to sepsis. *Shock*.

[88] Herzig D. S., Luan L., Bohannon J. K., Toliver-Kinsky T. E., Guo Y., Sherwood E. R. (2014). The role of CXCL10 in the pathogenesis of experimental septic shock. *Critical Care*.

[89] Kelly-Scumpia K. M., Scumpia P. O., Delano M. J. (2010). Type I interferon signaling in hematopoietic cells is required for survival in mouse polymicrobial sepsis by regulating CXCL10. *Journal of Experimental Medicine*.

[90] Cuenca A. G., Wynn J. L., Kelly-Scumpia K. M. (2011). Critical role for CXC ligand 10/CXC receptor 3 signaling in the murine neonatal response to sepsis. *Infection and Immunity*.

[91] Zhang X., Shen J., Man K. (2014). CXCL10 plays a key role as an inflammatory mediator and a non-invasive biomarker of non-alcoholic steatohepatitis. *Journal of Hepatology*.

[92] Elemam N. M., Talaat I. M., Maghazachi A. A. (2022). CXCL10 chemokine: a critical player in RNA and DNA viral infections. *Viruses*.

[93] Papić N., Krajinović V. (2021). Non-alcoholic fatty liver disease and sepsis. *Infektološki glasnik*.

[94] Abouelasrar Salama S., De Bondt M., De Buck M. (2020). Serum amyloid A1 (SAA1) revisited: restricted leukocyte-activating properties of homogeneous SAA1. *Frontiers in Immunology*.

[95] El Kebir D., József L., Khreiss T. (2007). Aspirin-triggered lipoxins override the apoptosis-delaying action of serum amyloid A in human neutrophils: a novel mechanism for resolution of inflammation. *Journal of Immunology*.

[96] Badolato R., Wang J. M., Murphy W. J. (1994). Serum amyloid A is a chemoattractant: induction of migration, adhesion, and tissue infiltration of monocytes and polymorphonuclear leukocytes. *Journal of Experimental Medicine*.

[97] Gouwy M., De Buck M., Pörtner N. (2015). Serum amyloid A chemoattracts immature dendritic cells and indirectly provokes monocyte chemotaxis by induction of cooperating CC and CXC chemokines. *European Journal of Immunology*.

[98] De Buck M., Berghmans N., Pörtner N. (2015). Serum amyloid A1*α* induces paracrine IL-8/CXCL8 via TLR2 and directly synergizes with this chemokine via CXCR2 and formyl peptide receptor 2 to recruit neutrophils. *Journal of Leukocyte Biology*.

[99] Migita K., Kawabe Y., Tominaga M., Origuchi T., Aoyagi T., Eguchi K. (1998). Serum amyloid A protein induces production of matrix metalloproteinases by human synovial fibroblasts. *Laboratory Investigation*.

[100] Gatt M. E., Urieli-Shoval S., Preciado-Patt L. (1998). Effect of serum amyloid A on selected in vitro functions of isolated human neutrophils. *Journal of Laboratory and Clinical Medicine*.

[101] Linke R. P., Bock V., Valet G., Rothe G. (1991). Inhibition of the oxidative burst response of *N*-formyl peptide-stimulated neutrophils by serum amyloid-A protein. *Biochemical and Biophysical Research Communications*.

[102] Webb N. R. (2021). High-density lipoproteins and serum amyloid A (SAA). *Current Atherosclerosis Reports*.

[103] Cheng N., Liang Y., Du X., Ye R. D. (2018). Serum amyloid A promotes LPS clearance and suppresses LPS-induced inflammation and tissue injury. *EMBO Reports*.

[104] Shah C., Hari-Dass R., Raynes J. G. (2006). Serum amyloid A is an innate immune opsonin for Gram-negative bacteria. *Blood*.

[105] Piotti K. C., Yantiss R. K., Chen Z., Jessurun J. (2016). Serum amyloid A immunohistochemical staining patterns in hepatitis. *Histopathology*.

[106] Wang Y., Huang H., Sun R. (2017). Serum amyloid a induces M2b-like macrophage polarization during liver inflammation. *Oncotarget*.

[107] S. Raju M., Kamaraju R. S., Sritharan V. (2016). Continuous evaluation of changes in the serum proteome from early to late stages of sepsis caused by *Klebsiella pneumoniae*. *Molecular Medicine Reports*.

[108] Linke R. P., Meinel A., Chalcroft J. P., Urieli-Shoval S. (2017). Serum amyloid A (SAA) treatment enhances the recovery of aggravated polymicrobial sepsis in mice, whereas blocking SAA’s invariant peptide results in early death. *Amyloid*.

[109] Sander L. E., Sackett S. D., Dierssen U. (2010). Hepatic acute-phase proteins control innate immune responses during infection by promoting myeloid-derived suppressor cell function. *Journal of Experimental Medicine*.

[110] Uhlar C. M., Whitehead A. S. (1999). Serum amyloid A, the major vertebrate acute-phase reactant. *European Journal of Biochemistry*.

[111] Karlsson M., Zhang C., Méar L. (2021). A single-cell type transcriptomics map of human tissues. *Science Advances*.

[112] Kristiansen M., Graversen J. H., Jacobsen C. (2001). Identification of the haemoglobin scavenger receptor. *Nature*.

[113] Jansma G., de Lange F., Kingma W. P. (2015). ‘Sepsis-related anemia’ is absent at hospital presentation; a retrospective cohort analysis. *BMC Anesthesiology*.

[114] Etzerodt A., Moestrup S. K. (2013). CD163 and inflammation: biological, diagnostic, and therapeutic aspects. *Antioxidants & Redox Signaling*.

[115] Ganz T. (2006). Hepcidin and its role in regulating systemic iron metabolism. *Hematology Am Soc Hematol Educ Program*.

[116] Crawford M. A., Ward A. E., Gray V. (2023). Disparate regions of the human chemokine CXCL10 exhibit broad-spectrum antimicrobial activity against biodefense and antibiotic-resistant bacterial pathogens. *ACS Infectious Diseases*.

[117] Heslop J. A., Rowe C., Walsh J. (2017). Mechanistic evaluation of primary human hepatocyte culture using global proteomic analysis reveals a selective dedifferentiation profile. *Archives of Toxicology*.

[118] Michailidis E., Vercauteren K., Mancio-Silva L. (2020). Expansion, in vivo-ex vivo cycling, and genetic manipulation of primary human hepatocytes. *Proceedings of the National Academy of Sciences of the United States of America*.

[119] Thursz M. R., Richardson P., Allison M. (2015). Prednisolone or pentoxifylline for alcoholic hepatitis. *New England Journal of Medicine*.

[120] Vergis N., Atkinson S. R., Knapp S. (2017). In patients with severe alcoholic hepatitis, prednisolone increases susceptibility to infection and infection-related mortality, and is associated with high circulating levels of bacterial DNA. *Gastroenterology*.

[121] Ray K. (2017). Alcoholic liver disease: alcoholic hepatitis: a warning for prednisolone and infection risk?. *Nature Reviews Gastroenterology & Hepatology*.

[122] Yeligar S. M., Chen M. M., Kovacs E. J., Sisson J. H., Burnham E. L., Brown L. A. S. (2016). Alcohol and lung injury and immunity. *Alcohol*.

[123] Louvet A., Wartel F., Castel H. (2009). Infection in patients with severe alcoholic hepatitis treated with steroids: early response to therapy is the key factor. *Gastroenterology*.

[124] Jenniskens M., Weckx R., Dufour T. (2018). The hepatic glucocorticoid receptor is crucial for cortisol homeostasis and sepsis survival in humans and male mice. *Endocrinology*.

[125] Zhang L., Rubins N. E., Ahima R. S., Greenbaum L. E., Kaestner K. H. (2005). Foxa2 integrates the transcriptional response of the hepatocyte to fasting. *Cell Metabolism*.

