# Supplementary material for: Cell-Specific Regulation of Inflammatory Cytokines and Acute-Phase Proteins by the Glucocorticoid Receptor
Source: Mediators Inflamm. 2023 Nov 28;2023:4399998. doi: 10.1155/2023/4399998 (PMC11606692; doi:10.1155/2023/4399998)
Supplement: Supplementary 1 — Figure S1: effects of glucocorticoid receptor (GR) on the reporter activities of FKBP5 intron5–6 in HepG2 cells and HEK293 cells. Figure S2: mRNA expression in healthy versus NASH livers. Figure S3: CXCL2 mRNA pre- and post-liver transplant. Figure S4: cytokine mRNA changes in human whole blood with LPS and DEX. Figure S5: approximation of chemokine lysate/media percentages. [file 4399998.f1.pdf]

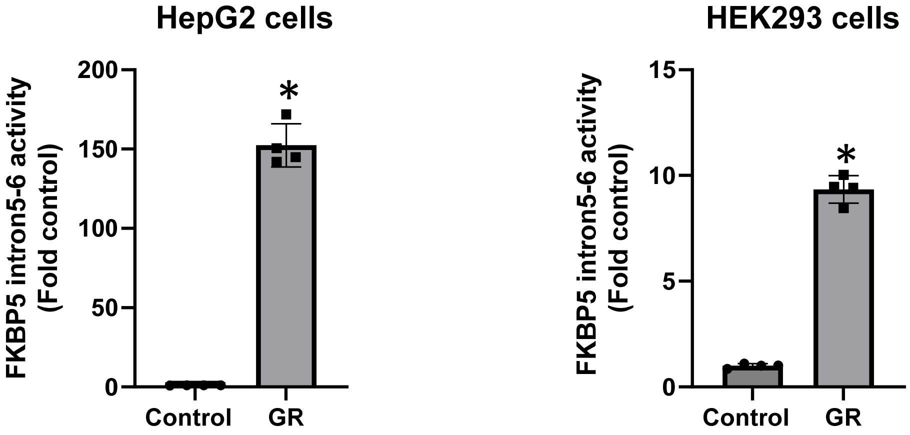


**Fig. S1. Effects of glucocorticoid receptor (GR) on the reporter activities of FKBP5 intron5-6 in HepG2 cells and HEK293 cells**. Cells in 96-well plate were co-transfected with pGL3-FKBP5 intron5-6, a secondary renilla luciferase reporter vector, and pk7-GR (20 ng), and treated with dexamethasone (10 nM for HepG2 and 100 nM for HEK293 cells). Dual-luciferase assay was conducted 24 h after transfection. N=4, mean ± SE. * $P < 0.05$ versus control. GR strongly activated FKBP5 intron5-6 in both HepG2 and HEK293 cells, with fold of activation higher in HepG2 cells than HEK293 cells.

## Hepatic mRNA expression in healthy humans versus NASH patients

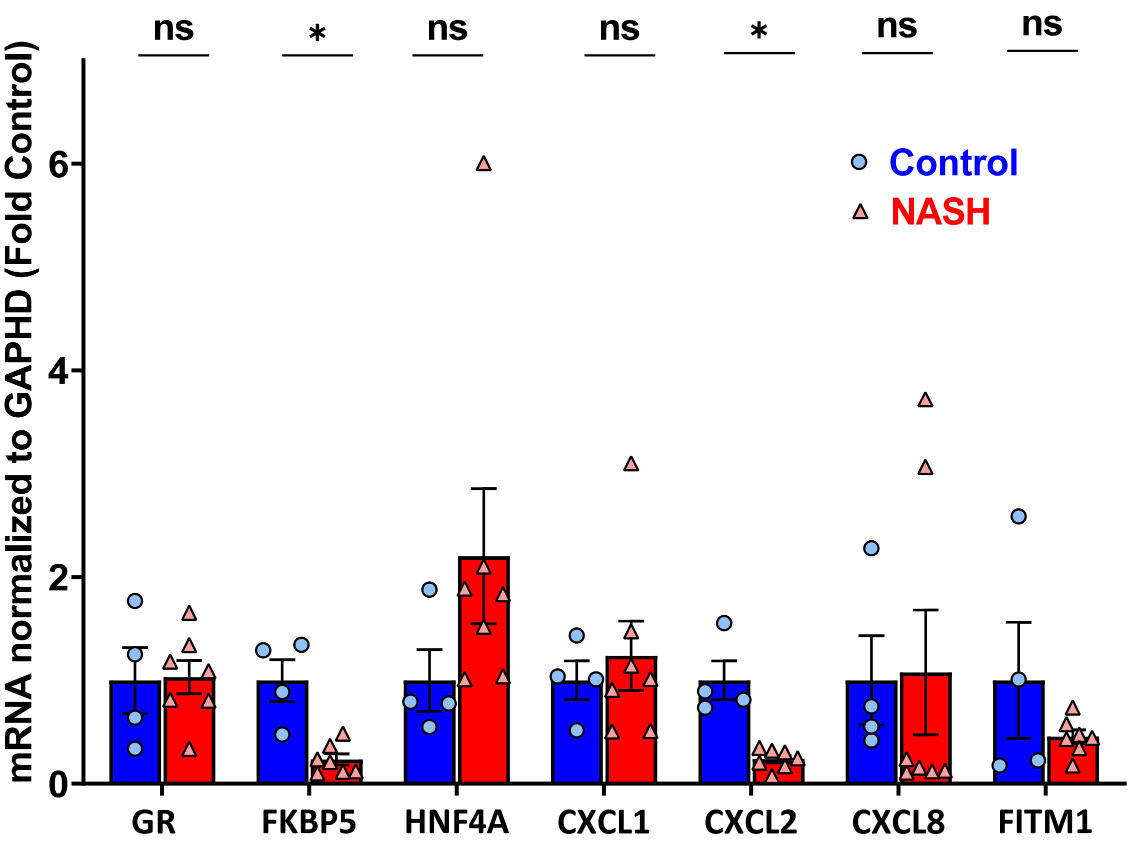


**Fig. S2. Hepatic mRNA expression in patients with and without nonalcoholic steatohepatitis (NASH).** Microarray data were retrieved from GEO DataSets (GSE17470). Mann-Whitney test was used for groups failing normality testing via Shapiro-Wilk test, Welch's t test was used for samples passing normality but with standard deviation discrepancies greater than 2-fold between control and NASH, and unpaired t test was used for samples with equal standard deviation and passing normality testing. n = 4-7 patients. * = $p \leq 0.05$, ** = $p \leq 0.01$

**Hepatic CXCL2 mRNA expression in patients pre- and post- liver transplantation**

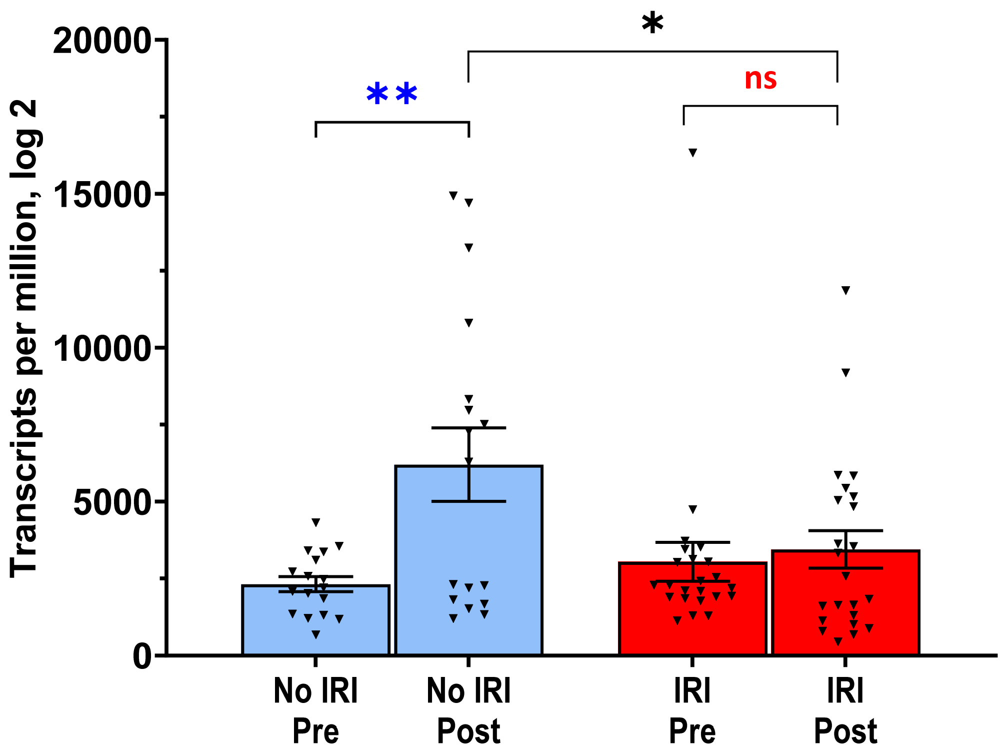


**Fig. S3. Hepatic CXCL2 mRNA expression in patients with or without ischemia reperfusion injury (IRI) pre- and post-liver transplantation.** RNA-sequencing data were retrieved from GEO DataSets (GSE151648). Wilcoxon matched-pairs signed rank test was used to compare matched pre-post transplant samples; Mann-Whitney test was used to compare non-IRI to IRI patients post transplant. * = $p \le 0.05$, ** = $p \le 0.01$

**Cytokine mRNA changes in human whole blood treated with LPS and DEX**

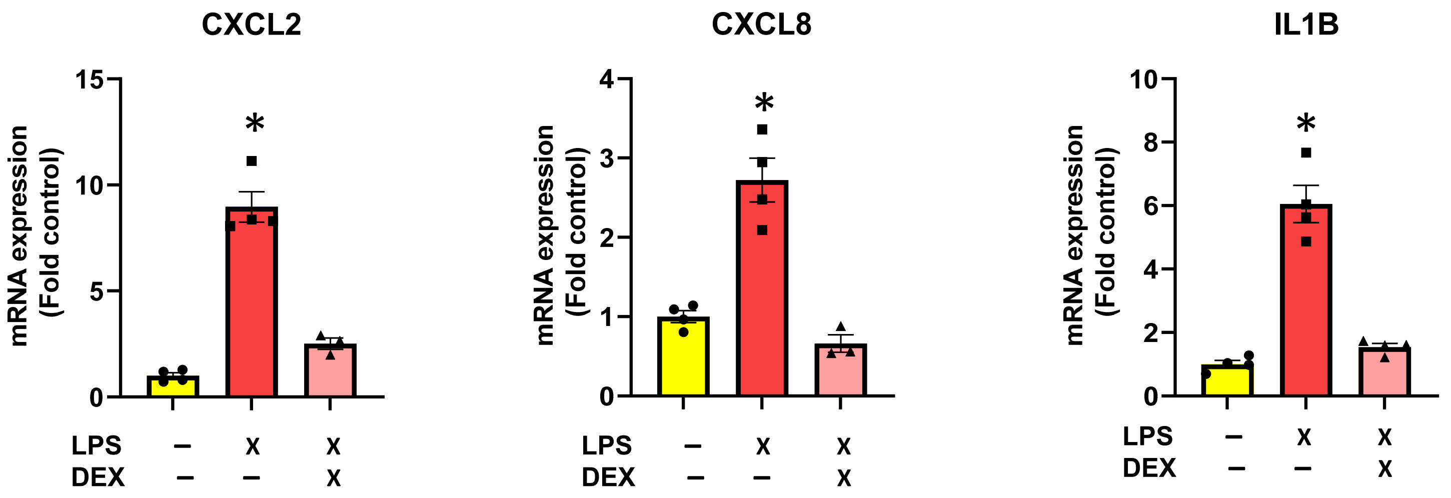


**Fig. S4. Effects of dexamethasone (DEX) on LPS-stimulated mRNA induction of cytokines in human whole blood.** Fresh heparinized human whole blood from healthy volunteer were added to 1:1 ratio of RPMI-1640 medium that contained 2 ng/mL LPS and/or 100 nM DEX and incubated for 4 h (at 37°C x 250 rpm). After 4 h incubation with LPS and/or DEX, total RNAs were prepared from blood cells for qPCR determination of mRNA expression, normalized to AKIRIN1. N = 4 replicates, mean ± SE. * $p < 0.05$ versus vehicle control using one-way ANOVA with Dunnett's multiple comparison.

**Approximation of total amounts and percentages of chemokines present in cell lysates and culture media**

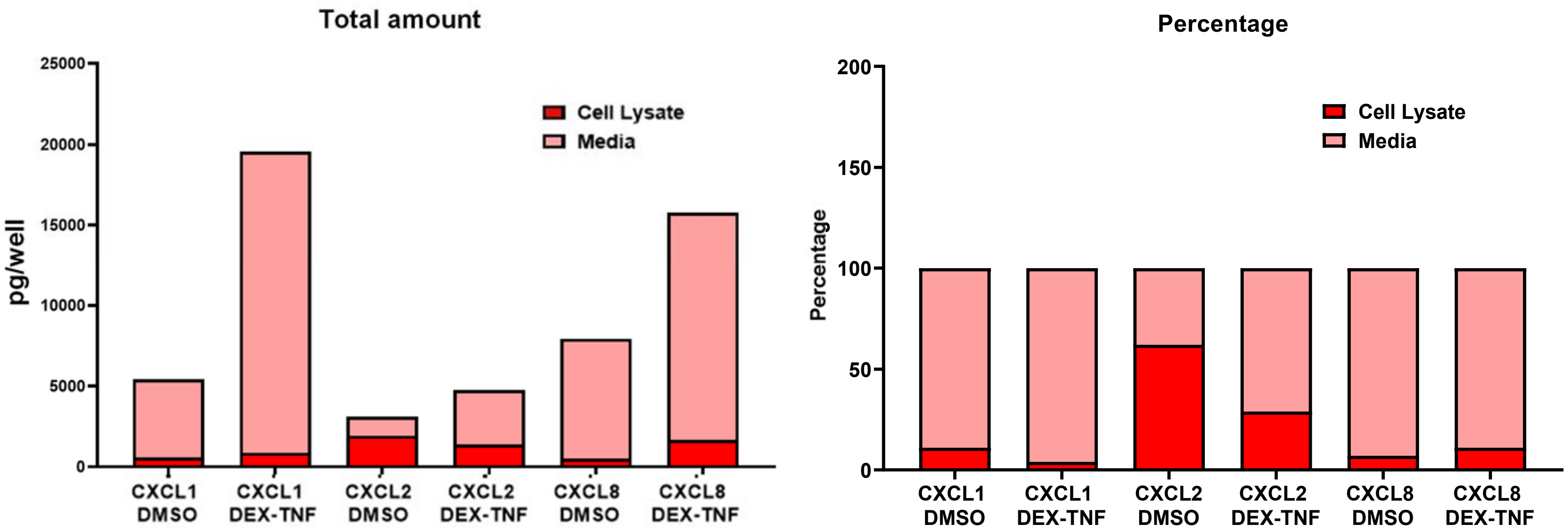


**Fig. S5. Preliminary approximation of total amounts and percentages of CXC chemokines present in cell lysates and culture media in primary human hepatocytes (PHH).** Cell lysates: Treated 1 well DMSO and 1 well DEX-TNF of 6-well plate (patient 1) with approximately 250-300 uL RIPA buffer with PPI. Vortexed several times. Digested at room temp and on ice for several minutes. Centrifuged 14000 g x10 minutes. Moved to new microcentrifuge tube. Diluted approximately 1:1 in more RIPA buffer. Concentration approximately 1.5 ug/uL protein by DC Protein Assay. Media assayed in a separate experiment using same or similarly treated wells.
