# Supplementary material for: Cell-Specific Regulation of Inflammatory Cytokines and Acute-Phase Proteins by the Glucocorticoid Receptor
Source: Mediators Inflamm. 2023 Nov 28;2023:4399998. doi: 10.1155/2023/4399998 (PMC11606692; doi:10.1155/2023/4399998)
Supplement: Supplementary 2 — Table S1: primers. Table S2: GRE sites predicted in the human CXCL2 promoter. [file 4399998.f2.pdf]

Supplemental Table 1: Primers

Primers for qPCR quantification of mRNA expression

hCXCL1_for	CCAGACCCGCCTGCTGAG
hCXCL1_rev	CCACGGACGCTCCTGCTG
hCXCL2_for	TGCAGGGAATTCACCTCAAG
hCXCL2_rev	CTGGTCAGTTGGATTTGCCAT
hCXCL8_for	GATTTCTGCAGCTCTGTGTGAA
hCXCL8_rev	AGACAGAGCTCTCTTCCATCA
hCXCL10_for	AGCAGAGGAACCTCCAGTCT
hCXCL10_rev	ATGCAGGTACAGCGTACAGT
hCCL2_for	GAAGCTGTGATCTTCAAGACC
hCCL2_rev	AGTCTTCGGAGTTTGGGTTTG
hIL1B_for	CCTGAGCTCGCCAGTGAAA
hIL1B_rev	GTGGTGGTCGGAGATTCGTA
hCD163_for	AAGACAGAGACAGCGGCTTG
hCD163_rev	CTTAAAGGCTGAACTCACTGG
hSAA1_for	CCAATTACATCGGCTCAGACA
hSAA1_rev	TGGATATTCTCTCTGGCATCG
hHAMP_for	CCTGACCAGTGGCTCTGTTT
hHAMP_rev	AGATGGGGAAGTGGGTGTCT
hPAI1_for	GCAAGGCACCTCTGAGAACT
hPAI1_rev	CACGTAGGATGGGGGATGGT

Primers for PCR cloning of reporter vectors for human CXC chemokine promoters

hCXCL1_Pro_KpnI	AAAAGGTACCGCGCAGGCTGCCACTCA
hCXCL1_Pro_MluI	AAAAACGCGTGGGGCTCAGCAGGCGGGTCT
hCXCL2_Pro_KpnI	AAAAAAGGTACCAGACCTCCTGAGCCCAACA
hCXCL2_Pro_MluI	AAAAAAACGCGTGTGCGAGGAGGAGAGCTG
hCXCL8_Pro_KpnI	GGAAGCGGTACCCTTGTTCTAACACCTGCCACT
hCXCL8_Pro_MluI	AAGGGGACGCGTGTTTACACACAGTGAGATGGTT
hCXCL10_Pro_KpnI	AAGTGCGGTACCCTTGCCAGTTCCAGATCTTTG
hCXCL10_Pro_MluI	AAGAAGACGCGTGGTGCTGAGACTGGAGGTTC

Primers for site-directed mutagenesis of CXCL2 promoters

CXCL2_Pro_407bpD_F	TAGATAAGACATGCTGCAGTTC
CXCL2_Pro_407bpD_R	TGTTGGGCTCAGGAGGTC
CXCL2_NFAT_Mut_F	CTCCGGTACCTCCAGCCCCAACCATGCATAAAA
CXCL2_NFAT_Mut_R	TCCAGGGCCAGCCTAATTCCCGGAGCTCCAGA
CXCL2_Pro_CreMut_F	TCAGACCCGGtaccCACTCGTGAGTGC
CXCL2_Pro_CreMut_R	CTGTCTTGCGTAACTCCC
CXCL2_HNF4_del_F	ACACTGAATTAGATCTTGGCAATGGTC
CXCL2_HNF4_del_R	TTATTTTAGTTAACAGTGTTGC

Supplemental table 2. GRE sequences, positions, and the dissimilarity scores of each GRE sequence in the 921 bp human CXCL2 promoter predicted by PROMO, with factor’s species limited to human and site’s species limited to human.

GRE Sequence	Position	Dissimilarity score
CAAAACA	-893	3.76%
CAAAAAA	-871	0.00%
CAAAGAG	-820	3.76%
ATATTTG	-661	7.53%
CAAAAGC	-623	8.97%
CAAACAT	-564	7.53%
